# TRAIP Mediates Alcohol‐Induced Liver Injury through Regulating β‐catenin Ubiquitin Degradation via Direct Interaction

**DOI:** 10.1002/advs.202521610

**Published:** 2026-09-11

**Authors:** Zhan Wu, Mingjiang Liu, Lijuan Liao, Zhaohong Mo, Wenli Xu, Linfeng Mao, Yubing Chen, Qiuli Xie, Ming Yao, Shilian Chen, Stephen Tomlinson, Sebastian Mueller, Songqing He, Guandou Yuan

**Affiliations:** ^1^ Division of Hepatobiliary Surgery The First Affiliated Hospital of Guangxi Medical University Nanning Guangxi China; ^2^ Key Laboratory of Early Prevention and Treatment for Regional High Frequency Tumor Ministry of Education Guangxi Medical University Nanning Guangxi China; ^3^ Guangxi Key Laboratory of Immunology and Metabolism for Liver Diseases Guangxi Medical University Nanning Guangxi China; ^4^ Department of Microbiology and Immunology Medical University of South Carolina Charleston South Carolina United States; ^5^ Center for Alcohol Research University Hospital Heidelberg Heidelberg Germany

**Keywords:** alcohol‐related liver disease, fatty liver disease, NIAAA, oxidative stress, TRAIP

## Abstract

Oxidative stress is a critical driver in the pathogenesis of alcohol‐related liver disease (ALD), promoting hepatic inflammation and injury. This study investigates the role of tumor necrosis factor receptor‐associated factor interacting protein (TRAIP) in ALD. We utilize in vivo ALD models with liver‐specific TRAIP overexpression (LSO) or knockout (LKO) mice. Chromatin immunoprecipitation and luciferase reporter assays confirm NF‐κB1 binding to the TRAIP promoter. In vitro, co‐immunoprecipitation and domain mapping with truncated mutants identify the interaction between the TRAIP coiled‐coil domain and the β‐catenin Armadillo domain. TRAIP is significantly upregulated in human and murine ALD tissues. Ethanol‐fed TRAIP^LSO^ mice exhibit heightened oxidative stress and inflammation but reduced steatosis, whereas TRAIP^LKO^ mice show the opposite. Mechanistically, ethanol‐induced NF‐κB1 activation transcriptionally upregulates TRAIP. TRAIP directly ubiquitinates β‐catenin in vitro and promotes its K48‐linked ubiquitination and proteasomal degradation in cells, independently of the canonical GSK3β/β‐TrCP pathway. Consequently, increased TRAIP suppresses the antioxidative response downstream of β‐catenin. The β‐catenin stabilizer SKL2001 effectively alleviates ethanol‐induced oxidative damage. We conclude that TRAIP promotes ALD by driving β‐catenin degradation and oxidative stress through a GSK3β/β‐TrCP‐independent mechanism, identifying the β‐catenin pathway and the compound SKL2001 as implicated in this process.

## Graphical abstract

This study reveals that NF‐κB1‐driven TRAIP upregulation in ALD correlates with disease severity. Mechanistically, TRAIP directly binds β‐catenin via its CC domain and promotes its K48‐linked ubiquitination and degradation, which is independent of the GSK3β/β‐TrCP pathway. This impairs the FOXO3‐mediated antioxidant response and exacerbates oxidative stress‐induced liver injury.

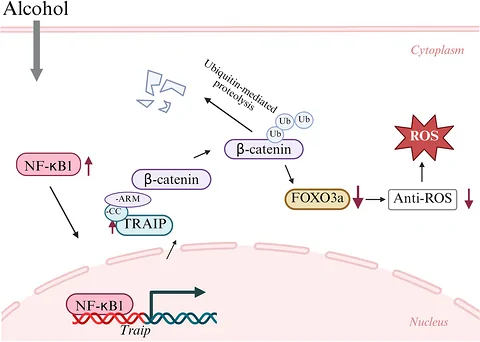

**Abbreviations**
AC: alcoholic cirrhosis
AH: alcoholic hepatitis
ALB: albumin
ALD: alcohol‐related liver disease
ALT: alanine aminotransferase
ARM: armadillo repeats
AST: aspartate aminotransferase
ATP: adenosine triphosphate
β‐catenin: beta‐catenin
β‐gal: β‐galactosidase
β‐TrCP: beta‐transducin repeat‐containing protein
CC: coiled coil
CD38: CD38 molecule
CD163: CD163 molecule
CHE: cholinesterase
ChIP: chromatin immunoprecipitation
Co‐IP: Co‐immunoprecipitation
CPT1a: carnitine palmitoyl transferase 1a
FASN: fatty acid synthase
FOXO3: forkhead box protein O3
F4/80: EGF‐like module‐containing mucin‐like hormone receptor‐like 1
4‐HNE: 4‐hydroxynonenal
GADD45a: growth arrest and DNA damage‐inducible protein alpha
GSH: glutathione
GSK3β: glycogen synthase kinase 3 beta
H&E: hematoxylin‐eosin staining
His: histidine
IL‐6: interleukin‐6
LAF: liquid alcohol feed
LV‐TRAIP: lentivirus‐TRAIP
Ly6G: lymphocyte antigen 6G
MELD: model for end‐stage liver disease
MDA: malondialdehyde
Mut: mutant
NF‐κB1: nuclear kappa B1
NIAAA: mouse model of chronic and binge ethanol feeding
PT: prothrombin time
RING: really interested new gene
TBIL: total bilirubin
TRAIP: tumor necrosis factor receptor associated factor interacting protein
TNF‐α: tumor necrosis factor alpha
TRAIP^LKO^
: liver‐specific knockout of TRAIP
ROS: reactive oxygen species
SOD: superoxide dismutase
SOD2: manganese superoxide dismutase 2
TF: transcription factor
TFBS: transcription factor binding site
TG: triglyceride
TRAIP^LSO^
: liver‐specific overexpression of TRAIP
Wnt: wingless‐type MMTV integration site family
WT: wild‐type

## 1 Introduction

Alcohol‐related liver disease (ALD) is one of the most prevalent chronic liver diseases worldwide [1], contributing to approximately 25% of liver cirrhosis‐related deaths in 2019 [2]. ALD encompasses a spectrum of liver conditions, ranging from simple steatosis to alcoholic hepatitis, fibrosis, cirrhosis, and end‐stage liver failure [3, 4]. Chronic excessive alcohol consumption induces hepatocyte injury, while acute binge drinking can result in extensive necrosis and hepatic decompensation [5, 6]. A key driver of ALD pathogenesis is oxidative stress, caused by excessive production of reactive oxygen species (ROS), which disrupts the hepatic redox balance [7, 8]. ROS trigger lipid peroxidation, forming cytotoxic byproducts that bind to DNA and promote apoptosis and autophagy [9]. Additionally, ROS and lipid peroxides activate Kupffer cells, leading to the release of pro‐inflammatory cytokines such as TNF‐α and IL‐6, which further exacerbate liver inflammation and hepatocellular injury [10].

Nuclear factor kappa B (NF‐κB) is a master regulator of inflammation and immune responses in ALD. Alcohol consumption significantly enhances NF‐κB activity, promoting its nuclear translocation and subsequent transcription of pro‐inflammatory genes [11]. ROS have been shown to be key mediators of NF‐κB activation, as inhibitors of NADPH oxidase and xanthine oxidase reduce ROS production and consequently suppress NF‐κB signaling [12, 13]. In contrast, the Wnt/β‐catenin signaling pathway plays a hepatoprotective role by counteracting oxidative stress‐induced apoptosis and mitigating ALD progression [14, 15]. Mice with hepatocyte‐specific β‐catenin deletion exhibit exacerbated oxidative stress and ethanol‐induced hepatocyte damage, highlighting β‐catenin's protective function in ALD [16]. Additionally, β‐catenin has been implicated in modulating hepatic lipid metabolism, with activation of the β‐catenin/FOXO3 antioxidant pathway conferring resistance to alcohol‐induced hepatic steatosis [17].

Tumor necrosis factor receptor‐associated factor‐interacting protein (TRAIP), an E3 ubiquitin ligase, was initially identified as a TNF receptor‐interacting protein [18, 19]. TRAIP contains a Really Interesting New Gene (RING) domain, responsible for its ubiquitin ligase activity, and coiled‐coil (CC) and leucine zipper domains that mediate protein‐protein interactions [20]. TRAIP plays essential roles in inflammation, apoptosis, cell cycle regulation, DNA damage repair, and chromosomal stability [20, 21, 22, 23]. Interestingly, we observed spontaneous hepatic lipid accumulation in aged mice with liver‐specific TRAIP overexpression (TRAIP^LSO^), suggesting that TRAIP is involved in hepatic lipid metabolism (Figure S3). Furthermore, we detected increased TRAIP expression in multiple ALD mouse models, leading us to hypothesize that TRAIP may modulate ALD pathogenesis by integrating inflammatory, oxidative, and metabolic pathways.

To explore this hypothesis, we analyzed TRAIP^LSO^ and liver‐specific TRAIP‐knockout (TRAIP^LKO^) mice and found that TRAIP exacerbates oxidative stress and inflammation while reducing hepatic steatosis. Mechanistically, TRAIP was transcriptionally activated by NF‐κB1 and promoted β‐catenin degradation, thereby impairing the β‐catenin/FOXO3 antioxidant pathway and worsening ethanol‐induced liver injury. These findings provide new insights into the TRAIP/β‐catenin axis as a target for ALD treatment, warranting further investigation into its clinical relevance and therapeutic applicability.

## 2 Results

### 2.1 TRAIP Is Highly Expressed in ALD

**FIGURE 1:**
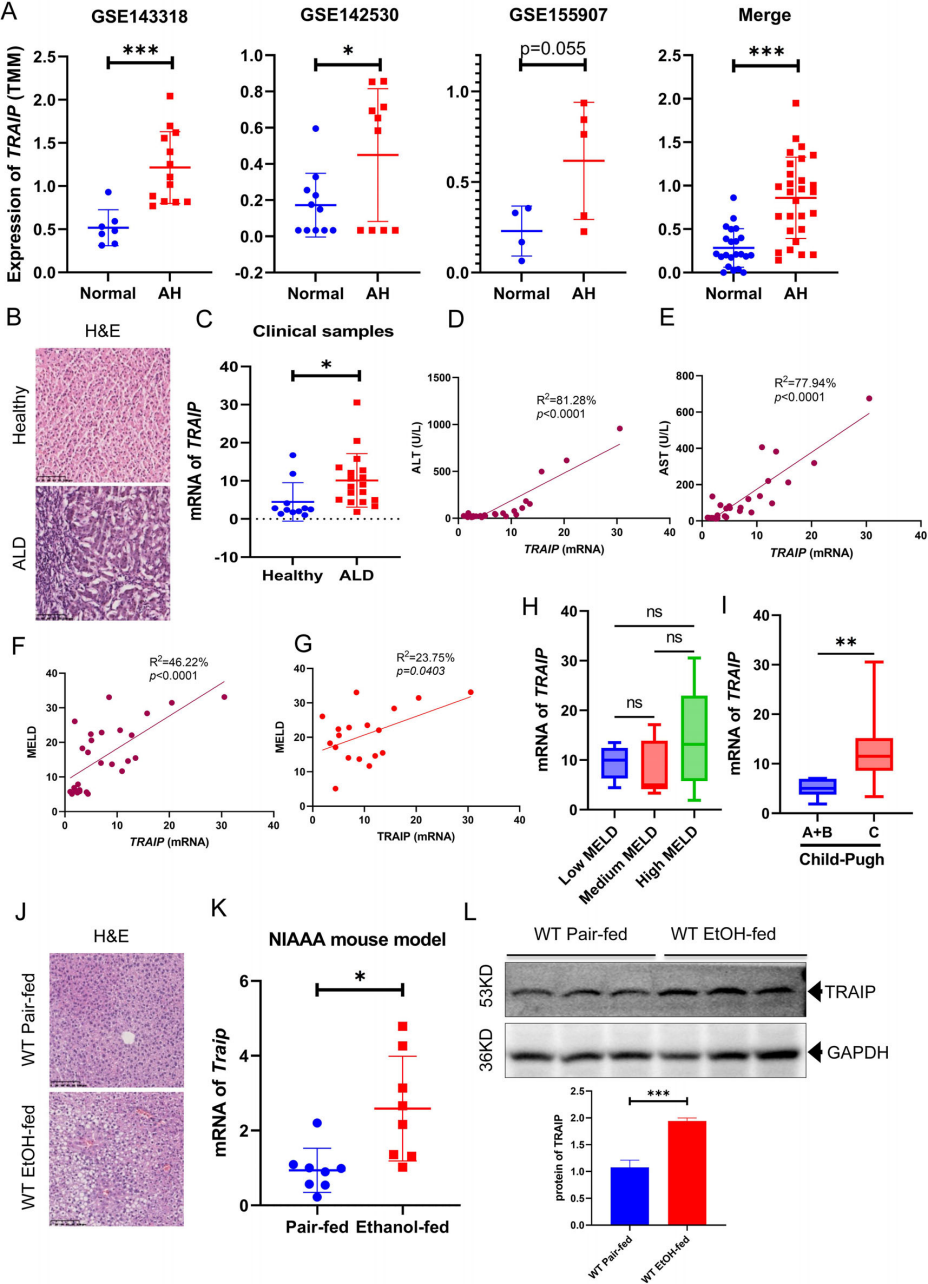
TRAIP is highly expressed in ALD. (A) *TRAIP* mRNA levels in human AH data from the GEO database (GSE143318, GSE142530, GSE155907, and their mergence, GSE143318 included 7 healthy controls and 13 AH samples. GSE142530 comprised 10 AH cases and 12 healthy controls. GSE155907 consisted of 5 AH cases and 4 healthy controls). (B) H&E staining of ALD clinical samples, Scale bar = 100 µm. (C) *TRAIP* mRNA levels in healthy individuals (Control, n = 11) and patients pathologically diagnosed with alcohol‐associated liver disease (ALD, n = 18). (D–F) Correlation analysis of *TRAIP* mRNA levels and ALT, AST and MELD levels in serum from healthy control (n = 9) and patients with ALD (n = 18) (Total n = 27). (G, H) Correlation analysis of *TRAIP* mRNA levels with MELD scores and *TRAIP* expression stratified by MELD score tertiles in ALD patients (n = 18). (I) *TRAIP* mRNA expression in ALD patients stratified by Child‐Pugh grade (A+B n = 6, C n = 12). (J) H&E staining of tissues from a mouse NIAAA model, Scale bar = 100 µm. (K) *Traip* mRNA levels in a mouse NIAAA model (n = 8/group). (L) TRAIP protein levels in a mouse NIAAA model (n = 3/group). Data represent mean ± SD, unpaired *t*‐test and one‐way ANOVA with Dunnett's post‐hoc test were used for group comparisons, spearman correlation was used for association analyses. ^*^ *p* < 0.05; ^**^ *p* < 0.01; ^***^ *p* < 0.001.

To determine whether TRAIP is involved in ALD progression, we first analyzed its expression in liver tissue samples from patients with AH using GEO databases, and found 4.37 times increase in *TRAIP* mRNA (Figure 1A). H&E staining of clinical ALD liver samples showed a significant increase in cell inflammatory infiltration (Figure 1B). Compared to normal liver tissue, *TRAIP* mRNA levels were significantly elevated in ALD tissue (Figure 1C). Across the spectrum from healthy liver donors to patients with AH and AC, elevated *TRAIP* mRNA levels correlated positively with disease severity markers (ALT, AST, MELD, TBIL) (Figure 1D–F and Figure S4D) and inversely with CHE (Figure S4F), in contrast to ALB (Figure S4E), to which it was not linked. To exclude the possibility that these correlations were driven by differences between healthy controls and ALD patients, we re‐analyzed the data restricted to ALD patients only. Within the ALD cohort, *TRAIP* mRNA expression positively correlated with MELD scores (Figure 1G). When ALD patients were stratified by MELD score tertiles, no significant difference in TRAIP expression was observed among the three groups (Figure 1H). TRAIP expression was significantly higher in Child‐Pugh C patients than in Child‐Pugh A and B patients (Figure 1I). Next, we constructed the typical NIAAA ALD mouse model in C57BL6J wild‐type mice. H&E staining of mouse livers showed significant fat deposition in the experimental group compared to the control group (Figure 1J). *Traip* mRNA and protein expression levels were significantly increased in the experimental group compared to the control group (Figure 1K,L). AML12 cells were stimulated with different concentrations of ethanol (50 mM, 100 mM, 200 mM) to confirm our in vivo findings in vitro. There was a dose‐dependent relationship between ethanol concentration and *Traip* mRNA and protein levels (Figure S4A,B). Similarly, TRAIP protein levels were elevated in ethanol‐treated HepG2 cells (Figure S4C).

### 2.2 TRAIP^LSO^ Exacerbates Oxidative Stress and Inflammatory Damage in Mouse Models of ALD

**FIGURE 2:**
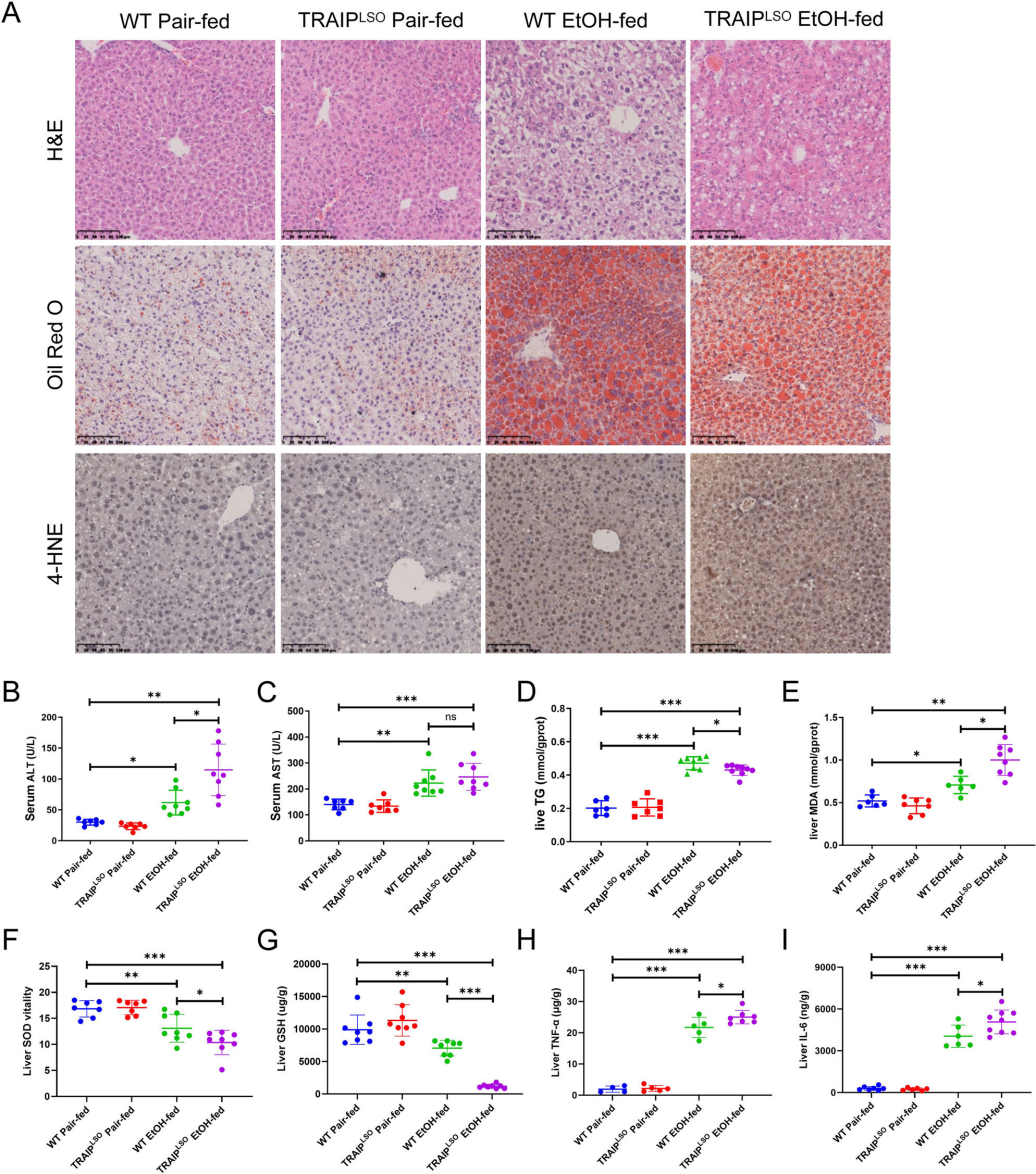
TRAIP^LSO^ exacerbates oxidative stress and inflammatory damage in mouse models of ALD. (A) Representative H&E and oil red O staining of livers from WT and TRAIP ^LSO^ mice in the NIAAA model, Scale bar = 100 µm. (B, C) Serum ALT and AST levels in WT and TRAIP ^LSO^ mice upon paired‐fed (n = 6–9 /group) or EtOH‐fed (n = 6–9/group) treatment. (D–G) Liver triglyceride (TG), MDA, SOD and GSH levels from WT and TRAIP ^LSO^ mice upon paired‐fed (n = 6–9/group) or EtOH‐fed (n = 6–9/group) treatment. (H, I) Liver TNF‐α and IL‐6 levels (n = 5–9/group). Data points identified as outliers were excluded. Data are presented as mean ± SD. P values were calculated by two‐way ANOVA. ^*^ *p* < 0.05, ^**^ *p* < 0.01, ^***^ *p* < 0.001, ns: not significant.

To further investigate the function of TRAIP in ALD, we exposed our TRAIP^LSO^ mice to alcohol. In the NIAAA model, H&E staining of liver sections from wild‐type mice fed with liquid alcohol diet (LAF) showed more obvious fat vacuoles compared to TRAIP^LSO^ mice fed with LAF. Also, oil red O staining showed more obvious lipid droplets, indicating more fat deposition in the liver (Figure 2A). 4‐HNE immunohistochemistry showed markedly stronger staining in the livers of TRAIP^LSO^ mice compared to wild‐type mice following alcohol exposure (Figure 2A). In the chronic feeding model, H&E staining showed similar results (Figure S5D), though not as significant as in the acute alcohol gavage model (Figure S5A). TRAIP^LSO^ mice fed with a LAF also showed a significant increase in ALT compared to wild‐type mice fed with a LAF (Figure 2B). There was no difference in ALT and AST levels between the two groups fed with liquid control feed (Figure 2B,C), suggesting that TRAIP^LSO^ itself did not affect liver function. The acute alcohol gavage model (Figure S5B,C) and the chronic model (Figure S5E,F) showed similar results. Analysis of liver homogenate suggested that the liver TG content of TRAIP^LSO^ mice fed with LAF was slightly reduced (Figure 2D). Compared with wild‐type mice fed with LAF, liver homogenate from TRAIP^LSO^ mice fed with the LAF showed a significant increase in MDA (Figure 2E), a significant decrease in SOD (Figure 2F) and GSH (2G). These findings suggest that TRAIP^LSO^ exacerbated oxidative stress damage in the livers of alcoholic liver mice. Similarly, the levels of TNF‐α and IL‐6 levels in the liver homogenates of TRAIP^LSO^ mice fed with the LAF were slightly higher than those of wild‐type mice fed with the LAF (Figure 2H,I), suggesting that TRAIP^LSO^ exacerbated an inflammatory response in a mouse model of alcoholic liver disease. Immunohistochemical analysis was further performed to assess neutrophil infiltration and macrophage polarization in the livers of TRAIP^LSO^ mice following alcohol exposure (Figure S6A). Ly6G staining, a marker for neutrophils, revealed significantly more positive cells in the livers of TRAIP^LSO^ mice fed with LAF compared to wild‐type mice. F4/80 staining, a marker for total macrophages, also showed increased positive areas in TRAIP^LSO^ mice. Furthermore, CD38 staining, an M1 polarization marker, was markedly elevated in TRAIP^LSO^ mice, while CD163 staining, an M2 polarization marker, was significantly reduced. These results indicate that TRAIP overexpression in hepatocytes promotes neutrophil infiltration and skews macrophage polarization toward a pro‐inflammatory M1 phenotype in ALD.

### 2.3 TRAIP^LKO^ Reduces Oxidative Stress and Inflammatory Damage a Mouse Model of ALD

**FIGURE 3:**
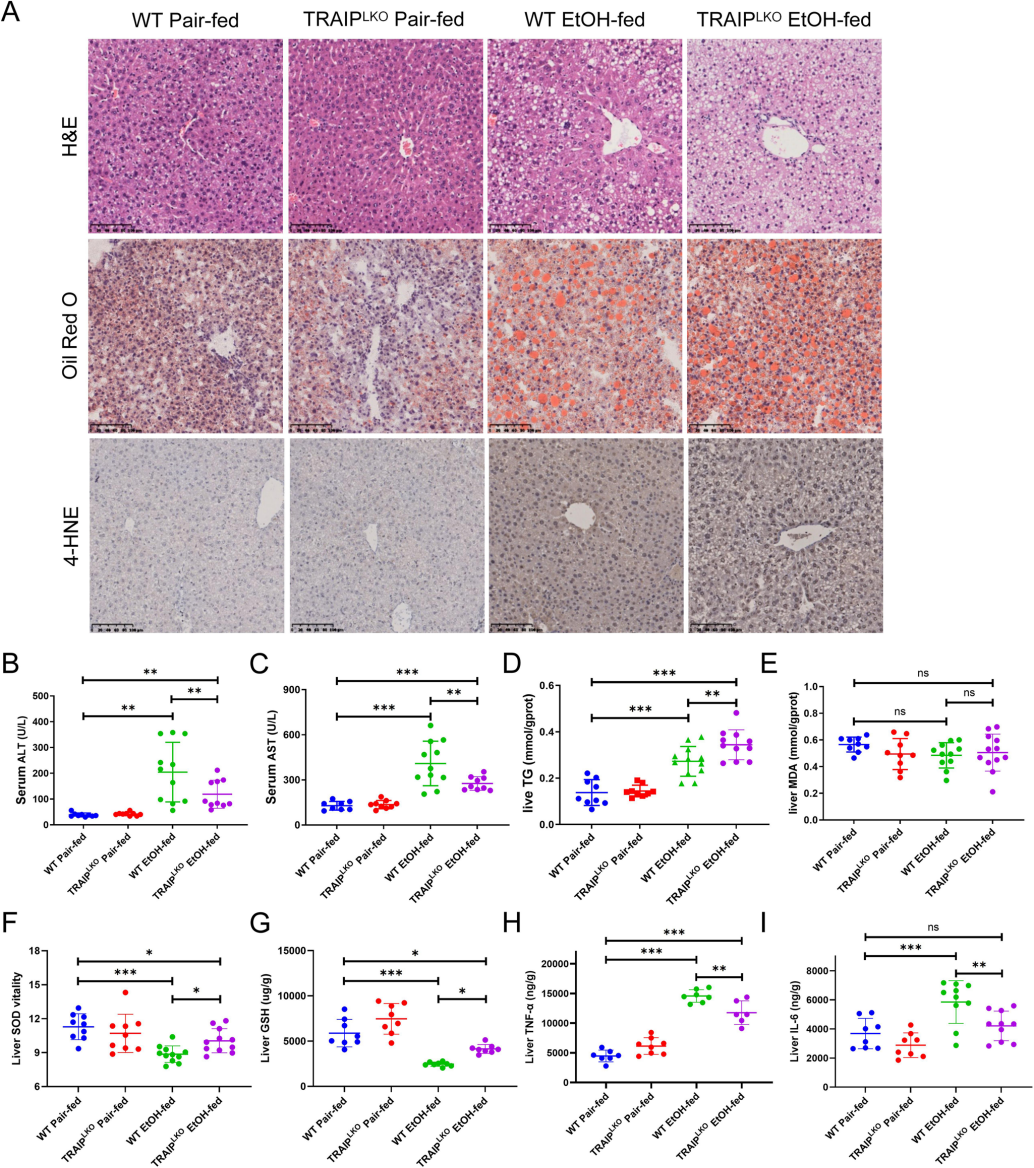
TRAIP^LKO^ reduces oxidative stress and inflammatory damage in a mouse model of ALD. (A) H&E and oil red O staining of livers from WT and TRAIP ^LKO^ mice in the NIAAA model, Scale bar = 100µm. (B, C) Serum ALT and AST levels(n = 8–11/group). (D–F) Liver triglyceride (TG), MDA, SOD and GSH levels from WT and TRAIP^LKO^ mice upon paired‐fed (n = 8–11/group) or EtOH‐fed (n = 8–11/group) treatment. (H, I) Liver TNF‐α and IL‐6 levels (n = 7–11/group). Data points identified as outliers were excluded. Data are presented as mean ± SD. P values were calculated by two‐way ANOVA. ^*^ *p* < 0.05, ^**^ *p* < 0.01, ^***^ *p* < 0.001, ns: not significant.

Next, in order to find out the impact of TRAIP deficiency on ALD, we treated TRAIP^LKO^ mice and their littermates in a protocol for chronic and binge ethanol feeding (NIAAA model). In contrast to TRAIP^LSO^ mice, in the NIAAA model, H&E staining of liver sections from TRAIP^LKO^ mice fed with the LAF showed more obvious fat vacuoles compared to wild‐type mice fed with the LAF, and corresponding lipid droplets were more pronounced, as detected by oil red O staining (Figure 3A). In contrast, 4‐HNE immunohistochemistry showed markedly weaker staining in the livers of TRAIP^LKO^ mice compared to wild‐type mice following alcohol exposure (Figure 3A). Compared with wild‐type mice fed with the LAF, TRAIP^LKO^ mice fed with the LAF showed a significant decrease in ALT and AST levels. There was no difference between the two groups fed with liquid control feed (Figure 3B,C), indicating that TRAIP^LKO^ itself did not affect liver function. The TG content in liver homogenates of TRAIP^LKO^ mice fed with the LAF was slightly increased (Figure 3D). The SOD and GSH level in the liver homogenates of TRAIP^LKO^ mice fed with the LAF was significantly increased compared to wild‐type mice fed with the LAF (Figure 3F,G), and there was no difference in MDA levels between the two groups (Figure 3E). These findings suggest that TRAIP^LKO^ alleviated oxidative stress damage in the alcoholic liver. The levels of TNF‐α and IL‐6 in liver homogenates of TRAIP^LKO^ mice fed with the LAF were significantly reduced compared to those seen in wild‐type mice fed with the LAF (Figure 3H,I), suggesting that TRAIP^LKO^ alleviated the inflammatory response in the alcoholic liver. Immunohistochemical analysis was further performed to assess neutrophil infiltration and macrophage polarization in the livers of TRAIP^LKO^ mice following alcohol exposure (Figure S6B). Ly6G staining revealed significantly fewer positive cells in the livers of TRAIP^LKO^ mice fed with LAF compared to wild‐type mice. F4/80 staining also showed decreased positive areas in TRAIP^LKO^ mice. Furthermore, CD38 staining, an M1 polarization marker, was markedly reduced in TRAIP^LKO^ mice, while CD163 staining, an M2 polarization marker, was significantly elevated. These results indicate that TRAIP deficiency in hepatocytes attenuates neutrophil infiltration and skews macrophage polarization toward an anti‐inflammatory M2 phenotype in ALD.

### 2.4 NF‐κB1 Activates Traip Transcription

**FIGURE 4:**
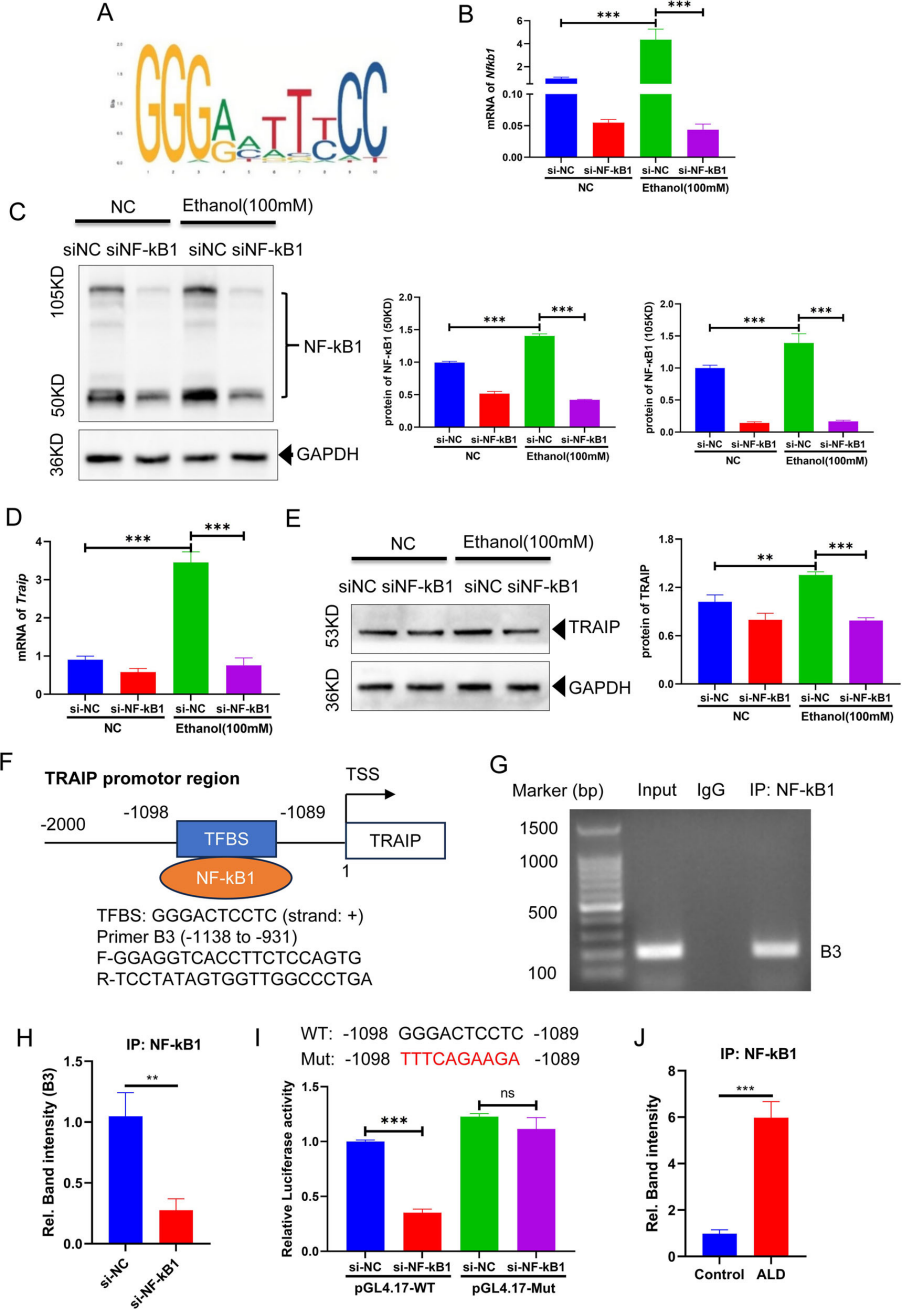
NF‐κB1 activates *Traip* transcription. (A) NF‐κB1 sequencing results. (B, C) The mRNA and protein levels of NF‐κB1 treated with siNF‐κB1 and ethanol (100mM) for 24h (n = 3/group) in AML12 cells. (D, E) TRAIP mRNA and protein levels treated with siNF‐κB1 and ethanol (100mM) for 24h (n = 3/group) in AML12 cells. (F) TRAIP promotor region and transcription factor binding site (TFBS). (G) *Traip* promoter DNA fragment pulled down by NF‐κB1. (H) ChIP‐qPCR treated with siNF‐κB1 (n = 3/group). (I) TFBS luciferase activity treated with siNF‐κB1 or pGL4.17‐Mut. (n = 3/group). (J) ChIP‐qPCR of ALD mice and controls (n = 3/group). Data are presented as mean ± SD. P values were calculated by one‐way ANOVA, two‐way ANOVA or unpaired *t*‐test as appropriate. ^**^ *p* < 0.01, ^***^ *p* < 0.001, ns: not significant.

Using the JASPAR database, we predicted that NF‐κB1 may be a transcriptional activator of *Traip* (Figure 4A and Figure S7A). In AML12 cells treated with ethanol, NF‐κB1 mRNA and protein levels were significantly increased (Figure S7B,C). A consistent upregulation of NF‐κB1 protein was also confirmed in ethanol‐treated HepG2 cells (Figure S7D) and in EtOH‐fed WT mice (Figure S7E). siRNA‐mediated silencing of NF‐κB1 (Figure S7F,H) significantly reduced *Traip* mRNA levels (Figure S7G). Under the combined treatment of ethanol and NF‐κB1, the mRNA and protein levels of NF‐κB1 were significantly reduced due to siRNA‐mediated silencing (Figure 4B,C). Correspondingly, the silencing of NF‐κB1 also led to a marked downregulation of TRAIP at both the mRNA and protein levels (Figure 4D,E). The JASPAR database predicted the TFBS of *Traip* to be between −1098 and −1089 bp, containing the sequence GGGACTCCTC (Figure 4F). Chromatin immunoprecipitation suggested that NF‐κB1 binds to the TFBS fragment of *Traip* (Figure 4G and Figure S7I,J). In addition, silencing NF‐κB1 significantly reduced the amount of DNA bound to the TFBS (Figure 4H). Furthermore, silencing NF‐κB1 decreased TFBS luciferase activity, and following mutation of the TFBS sequence, silencing NF‐κB1 no longer affected its luciferase activity (Figure 4I). To verify the in vivo relevance of the NF‐κB1→*Traip* transcriptional axis, the mouse NIAAA model was established in conjunction with the administration of the NF‐κB1‐specific inhibitor EN450. H&E staining revealed that liver injury was significantly alleviated in the group treated with EN450 compared to the standard model group (Figure S7K), accompanied by markedly reduced serum levels of ALT and AST (Figure S7L,M). Consistently, a concomitant reduction in both mRNA and protein levels of TRAIP was observed in the EN450‐treated group compared to the standard model group (Figure S7N,O). To assess whether NF‐κB1 occupies the *Traip* promoter in vivo, ChIP‐qPCR was performed on liver tissues from ALD mice and controls. NF‐κB1 occupancy at the *Traip* promoter was significantly higher in ALD mice than in controls (Figure 4J), indicating that NF‐κB1 directly binds to the *Traip* promoter in the liver under pathological conditions.

### 2.5 TRAIP Interacts Directly with β‐Catenin

**FIGURE 5:**
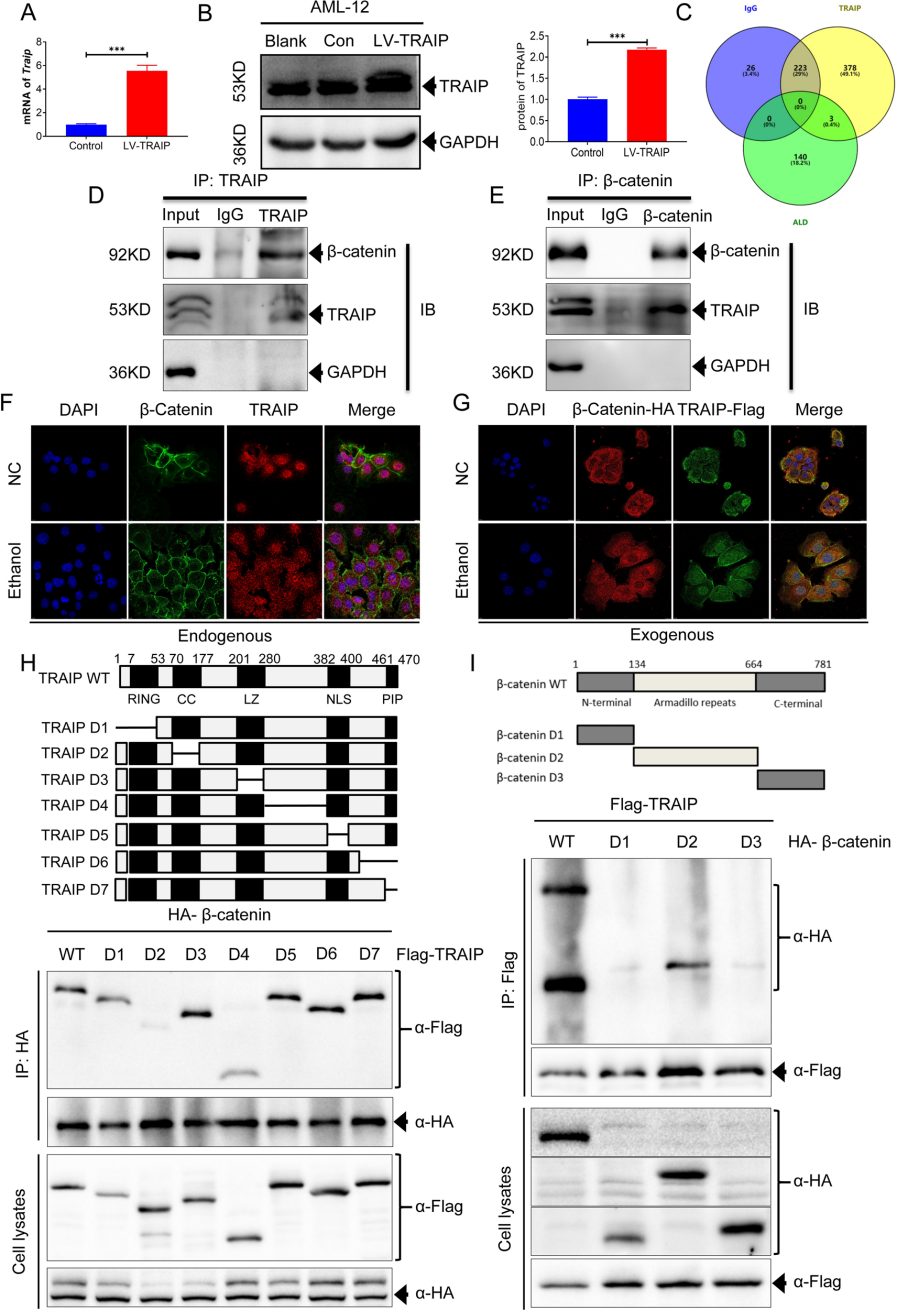
TRAIP interacts with β‐catenin. (A, B) The mRNA and protein levels of TRAIP in AML12 cells after infection with a TRAIP‐expressing lentivirus. (n = 3/group). (C)Proteins pulled down by TRAIP that are involved in ALD. (D, E) Co‐IP of TRAIP and β‐catenin (n = 3/group). (F, G) Representative immunofluorescence staining of endogenous and exogenous β‐catenin and TRAIP in ETOH‐treated AML12 cells. (H) Different TRAIP mutants subjected to Co‐IP with the full length of β‐catenin. (I) Different β‐catenin mutants subjected to Co‐IP with the full length of TRAIP. Amino acid positions and expected molecular weights of the truncation mutants are provided in Table S3. Data are presented as mean ± SD. p‐values were calculated using unpaired *t*‐test. ^*^ *p* < 0.05, ^***^ *p* < 0.001.

Infecting AML12 cells with a lentivirus expressing TRAIP resulted in stable TRAIP overexpression in AML12 cell lines. The mRNA and protein levels of TRAIP were significantly increased compared to the control group (Figure 5A,B), indicating successful cell line construction. Mass spectrometry analysis of the co‐immunoprecipitated proteins showed that 604 proteins were pulled down by TRAIP, 249 of which were also pulled down by IgG. A total of 143 ALD pathway proteins were retrieved from the KEGG database and compared with the 355 unique TRAIP‐binding proteins. In total, three proteins overlapped between TRAIP‐binding proteins and ALD pathway proteins: β‐catenin, FASN, and CPT1a (Figure 5C). Based on the three indicators of Protein Score, Unique Peptides, and Abundance, β‐catenin was selected for further study. The TRAIP antibody pulled down β‐catenin (Figure 5D), and a β‐catenin antibody pulled down TRAIP (Figure 5E), suggesting that the two proteins interact with each other. Immunolocalization showed that endogenous β‐catenin was mainly concentrated in the cell membrane and cytoplasm, while TRAIP is mainly concentrated in the cytoplasm and nucleus. Ethanol stimulation increased the cytoplasmic content of TRAIP (Figure 5F). Exogenous HA‐β‐catenin was mainly concentrated in the cell membrane and cytoplasm, while Flag tagged TRAIP was mainly concentrated in the cytoplasm and nucleus. Ethanol stimulation increased the cytoplasmic content of both (Figure 5G). To map the domains potentially involved in the TRAIP–β‐catenin interaction, we generated a series of TRAIP and β‐catenin truncation mutants. Co‐immunoprecipitation assays revealed that Flag‐TRAIP D2, which lacks the CC domain, failed to be pulled down by full‐length HA‐β‐catenin (Figure 5H). Conversely, full‐length TRAIP efficiently bound to HA‐β‐catenin D2, which contains the ARM domain, but not to HA‐β‐catenin D1 or D3 (Figure 5I). These data suggest that the CC domain of TRAIP is involved in binding to the ARM domain of β‐catenin. The peptide segment of β‐catenin detected by mass spectrometry is LLNDEDQVVVNK (Figure S8A,B), which represents amino acids 159–170, located precisely within the ARM region of β‐catenin.

### 2.6 TRAIP Directly Ubiquitinates β‐Catenin Independent of GSK3β/β‐TrCP

**FIGURE 6:**
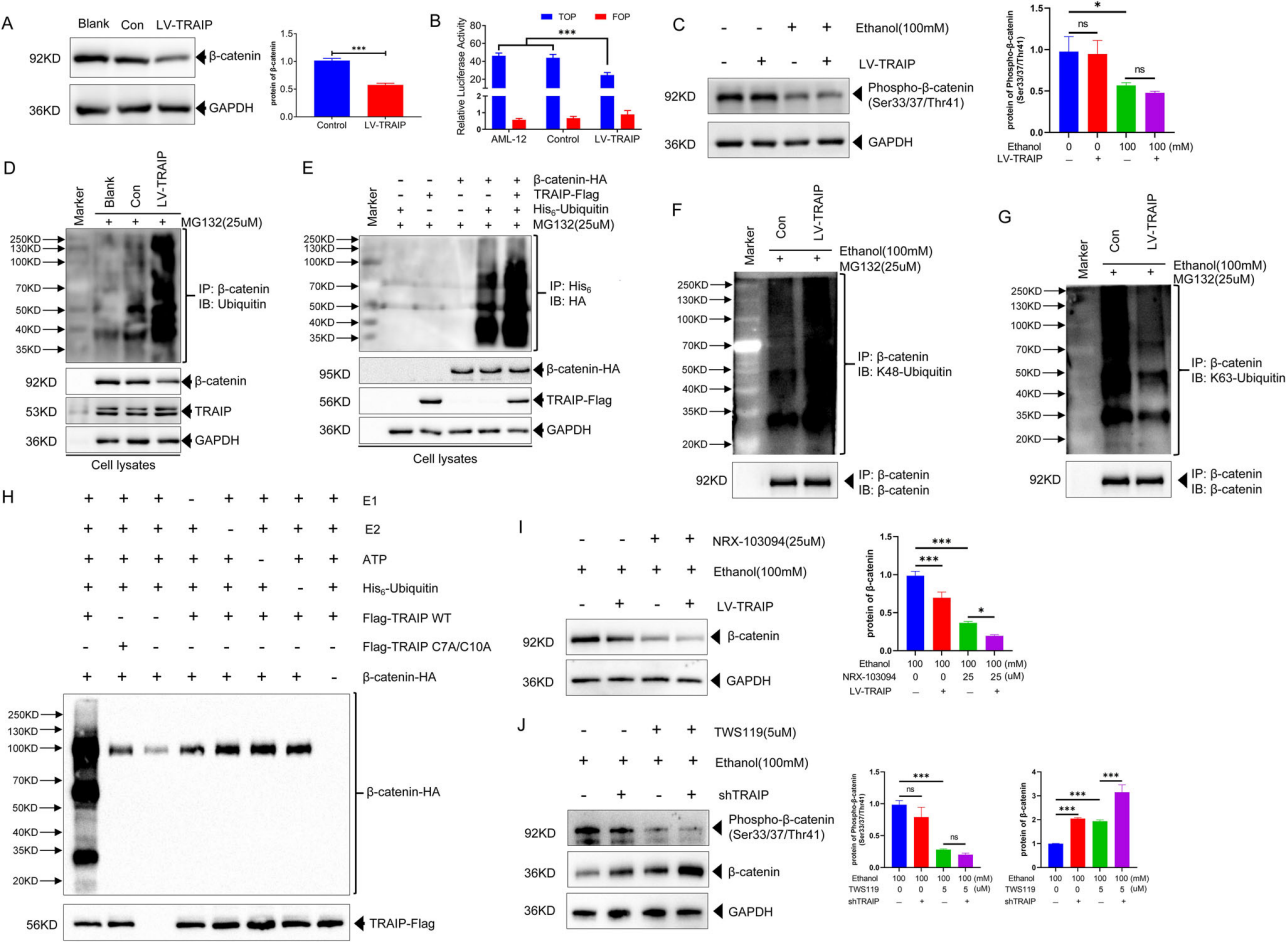
TRAIP directly targets β‐catenin for GSK3β/β‐TrCP‐independent K48‐linked ubiquitination. (A) β‐catenin protein levels in LV‐TRAIP AML12 cells (n = 3/group). (B) TOP/FOP luciferase activity in Control and LV‐TRAIP AML12 cells (n = 3/group). (C) Phospho‐β‐catenin (Ser33/37/Thr41) levels in LV‐TRAIP or ethanol (100mM)‐treated AML12 cells for 24h (n = 3/group). (D, E) Endogenous ubiquitination (D, n = 3/group) and Exogenous ubiquitination (E, n = 3/group) assay. (F) Analysis of K48‐linked polyubiquitination of β‐catenin. Lysates from Control and LV‐TRAIP AML12 cells treated with ethanol (100 mM) and MG132 (25 µM) for 24h. The bottom panel shows immunoprecipitated β‐catenin (92 kDa) as a loading control (n = 3/group). (G) Parallel assessment of K63‐linked ubiquitination under identical treatment conditions. IP/IB analysis was performed as in (F), but using an anti‐K63 ubiquitin antibody to detect K63‐linked ubiquitination (n = 3/group). (H) TRAIP directly ubiquitinates β‐catenin in a cell‐free system. In vitro ubiquitination assays were performed using the indicated purified components: E1, E2 (UbcH5c), ATP, His‐ubiquitin, β‐catenin‐HA as substrate, and Flag‐TRAIP (wild‐type or RING domain mutant C7A/C10A). Reaction mixtures were incubated at 37°C for 2 h, followed by immunoblotting with anti‐HA antibody to detect β‐catenin ubiquitination (top panel). The ubiquitination ladder indicates polyubiquitination of β‐catenin. Input levels of TRAIP were confirmed with anti‐Flag antibody (bottom panel). Representative images from three independent experiments are shown. (I) β‐catenin protein levels in ethanol‐treated Control and LV‐TRAIP AML12 cells combine with NRX‐103094(β‐ TrCP agonist)) for 24h (n = 3/group). (J) Phospho‐β‐catenin (Ser33/37/Thr41) and β‐catenin protein levels in ethanol‐treated Control and shTRAIP AML12 cells combine with TWS119 (GSK3β inhibitor) for 24h (n = 3/group). Data are presented as mean ± SD. P values were calculated by one‐way ANOVA, two‐way ANOVA or unpaired *t*‐test as appropriate. ^*^ *p* < 0.05, ^***^ *p* < 0.001, ns: not significant.

Given the direct interaction between TRAIP and β‐catenin, we next sought to explore how the two molecules interact. First, in ethanol‐treated AML12 cells, β‐catenin protein levels were significantly reduced (Figure S9A). Consistently, ethanol treated HepG2 cells (Figure S9B) and ethanol‐fed mice with ALD also exhibited a significant decrease in β‐catenin protein levels (Figure S9C). Overexpression of TRAIP (LV‐TRAIP) in AML12 cells led to a decrease in β‐catenin protein levels (Figure 6A) and a reduction in TOP/FOP luciferase activity (Figure 6B), while interestingly, the phosphorylation level of β‐catenin remained unaffected (Figure 6C). In both ethanol‐treated AML12 cells and alcohol‐fed WT mice, GSK3β/β‐TrCP were significantly upregulated (Figure S9E,F), leading to the activation of the canonical β‐catenin degradation pathway. TRAIP is an E3 ubiquitination ligase, and binding of its CC terminal to β‐catenin reduced its protein level and pathway activity. Therefore, we speculated that TRAIP may degrade β‐catenin through ubiquitination. LV‐TRAIP increased the ubiquitination level of endogenous β‐catenin (Figure 6D and Figure S9G), while the addition of exogenous Flag tagged TRAIP resulted in a significant increase in ubiquitinated HA‐β‐catenin (Figure 6E and Figure S9H). Compared with full‐length TRAIP, the ubiquitination levels in cells expressing TRAIP lacking the RING and CC domains were significantly reduced (Figure S9I). To further delineate the type of ubiquitin linkage mediated by TRAIP, we measured the levels of K48‐ and K63‐linked ubiquitination on β‐catenin. Our results indicated that LV‐TRAIP significantly increased K48‐linked ubiquitination (Figure 6F and Figure S9J) while decreasing K63‐linked ubiquitination (Figure 6G and Figure S9K). To determine whether TRAIP directly catalyzes β‐catenin ubiquitination in the absence of other cellular components, we performed cell‐free in vitro ubiquitination assays using purified E1, E2, ATP, His_6_‐Ubiquitin, and recombinant Flag‐TRAIP and HA‐β‐catenin proteins. Recombinant TRAIP‐Flag directly ubiquitinated β‐catenin, whereas the C7A/C10A amino acid mutant in the RING domain lost this activity (Figure 6H), confirming that TRAIP functions as a direct E3 ligase for β‐catenin. To determine whether the Flag‐TRAIP C7A/C10A mutant lost ubiquitination activity due to impaired β‐catenin binding, co‐IP assays were performed. The mutant retained its interaction with β‐catenin (Figure S9L,M). To further assess the physiological requirement of TRAIP E3 ligase activity, rescue experiments were performed in TRAIP^LKO^ primary hepatocytes. Re‐expression of TRAIP WT, but not the C7A/C10A mutant, restored β‐catenin degradation (Figure S9N). Consistently, TRAIP WT, but not the C7A/C10A mutant, also restored ROS levels (Figure S9O). To further determine if TRAIP‐mediated ubiquitination and degradation of β‐catenin differs from the canonical GSK3β/β‐TrCP pathway, a dual strategy of LV‐TRAIP overexpression and β‐TrCP agonist (NRX‐103094) application was utilized. Having confirmed the efficacy of NRX‐103094 (Figure S9P), we found that its combination with LV‐TRAIP induced a more pronounced reduction in β‐catenin levels than either treatment alone (Figure 6I). Similarly, a dual strategy of shTRAIP knockdown and GSK3β inhibitor (TWS119) application was utilized. We found that its combination with shTRAIP induced a more pronounced increase in β‐catenin levels than either treatment alone (Figure 6J). These data demonstrate that TRAIP and the canonical pathway operate in parallel to regulate β‐catenin stability.

### 2.7 TRAIP Mediates β‐Catenin/FOXO3 Antioxidant Stress Signaling Pathway

**FIGURE 7:**
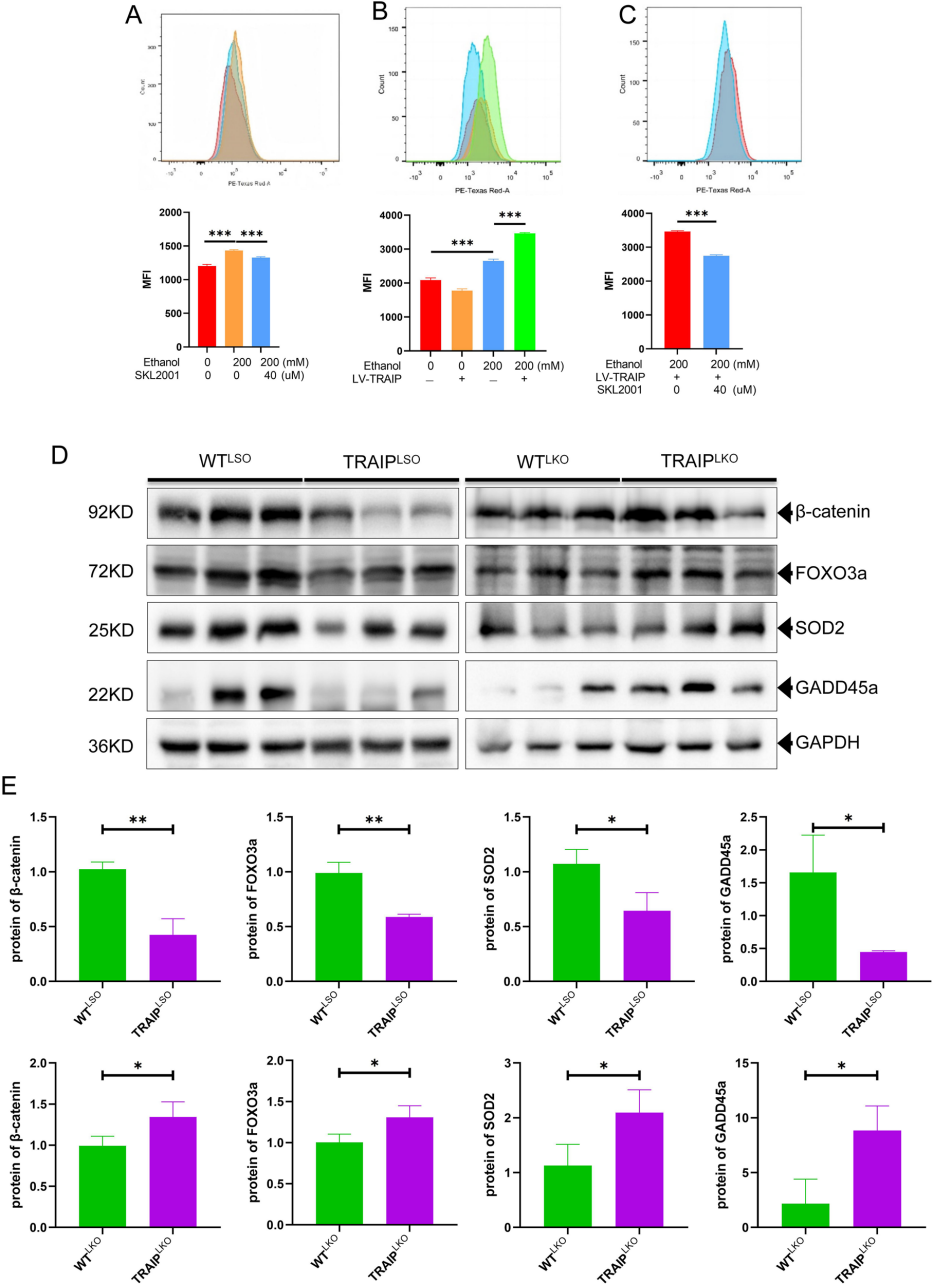
TRAIP mediates β‐catenin/FOXO3 antioxidant stress signaling pathway. (A) ROS activity levels in AML12 cells treated with ethanol (200mM) and SKL2001 (β‐catenin agonist) for 24h (n = 3/group). (B, C) ROS activity levels in LV‐TRAIP AML12 cells treated with ethanol (200mM) and SKL2001 (n = 3/group) for 24h. (D) Antioxidant stress‐related protein levels in TRAIP^LSO^ and TRAIP^LKO^ mice with EtOH‐fed (β‐catenin, FOXO3a, SOD2, GADD45a). (E) Densitometric quantification of antioxidant stress‐related protein levels normalized to GAPDH in TRAIP^LSO^ and TRAIP^LKO^ mice with EtOH‐fed (β‐catenin, FOXO3a, SOD2, GADD45a) (n = 3/group). Data are presented as mean ± SD. P values were calculated by one‐way ANOVA, two‐way ANOVA or unpaired *t*‐test as appropriate. ^*^ *p* < 0.05, ^**^ *p* < 0.01, ^***^ *p* < 0.001.

Ethanol treatment significantly increased ROS levels in AML12, HepG2 cells and primary hepatocytes, treatment with β‐catenin agonist SKL2001 resulted in a decrease in ROS levels (Figure 7A and Figure S10A). Compared to control AML12 cells treated with ethanol, LV‐TRAIP AML12 cells showed significantly higher ROS levels upon ethanol treatment (Figure 7B), and SKL2001 treatment reduced ROS levels in ethanol‐treated LV‐TRAIP AML12 cells (Figure 7C and Figure S10B,C). In our experimental system, overexpression of TRAIP was associated with reduced protein levels of β‐catenin, FOXO3a, SOD2, and GADD45a, all key components of the antioxidant stress pathway, whereas knocking out TRAIP led to increased levels of these proteins (Figure 7D,E).

### 2.8 SKL2001 Alleviates Liver Oxidative Stress Damage in ALD Mice

**FIGURE 8:**
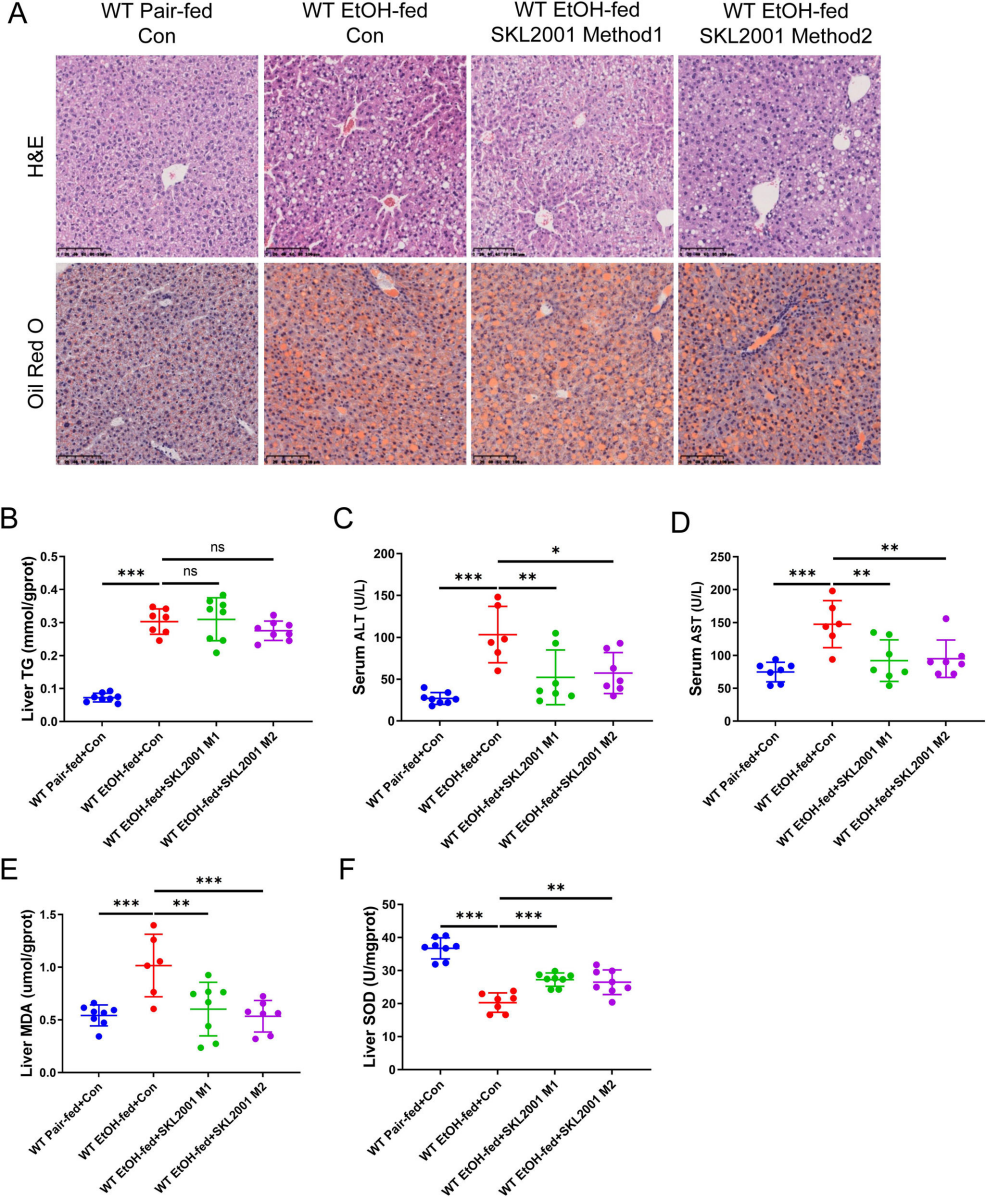
SKL2001 alleviates liver oxidative stress damage in a mouse model of ALD. (A) H&E and oil red O staining of livers from WT and WT+SKL2001 mice in the NIAAA model, Scale bar = 100µm. (B) Liver TG levels in WT and WT+SKL2001 mice upon paired‐fed (n = 6–8 /group) or EtOH‐fed (n = 6–8/group) treatment. (C, D) Serum ALT and AST levels in WT and WT+SKL2001 mice upon paired‐fed (n = 6–8 /group) or EtOH‐fed (n = 6–8/group) treatment. (E, F) Liver MDA levels and SOD activity in WT and WT+SKL2001 mice upon paired‐fed (n = 6–8/group) or EtOH‐fed (n = 6–8/group) treatment. P values were calculated by one‐way ANOVA. ^**^ *p* < 0.01, ^***^ *p* < 0.001, ns: not significant.

SKL2001 is a novel agonist of the Wnt/β‐catenin signaling pathway [24]. It stabilizes intracellular β‐catenin by disrupting the interaction between Axin and β‐catenin, thereby upregulating β‐catenin transcription and inhibiting β‐catenin phosphorylation at specific sites, preventing its degradation. Considering this mechanism of action, we treated mice with SKL2001 to determine whether intervention with the TRAIP target protein β‐catenin is effective. We found that there was no significant difference between H&E stained and oil red O stained liver sections from wild‐type mice fed with the LAF and treated with SKL2001, and wild‐type mice fed with the LAF and left untreated (Figure 8A). There was also no statistically significant difference in the TG content of liver homogenates from the experimental group and the control group (Figure 8B). Nevertheless, wild‐type mice fed with the LAF and treated with SKL2001 showed significant reductions in ALT and AST levels when compared to wild‐type mice fed with the LAF (Figure 8C,D). Compared to wild‐type mice fed with the LAF, wild‐type mice fed with the LAF and treated with SKL2001 also showed a significant decrease in MDA and a significant increase in SOD in the liver homogenates (Figure 8E,F). These findings suggest that SKL2001 alleviated oxidative stress damage in the liver of alcoholic liver mice.

We further treated NIAAA model TRAIP^LSO^ mice with SKL2001 and found that serum ALT and AST levels were significantly reduced in TRAIP^LSO^ mice treated with SKL2001 (Figure S11B,C). This demonstrates the beneficial effects of targeting β‐catenin in a mouse model of ALD.

## 3 Discussion

ALD remains a leading cause of morbidity and mortality worldwide, with oxidative stress serving as a central driver of its pathogenesis [1, 2, 4, 7]. In this study, we identified TRAIP as a contributing factor in ethanol‐induced hepatic oxidative stress and inflammation through its interaction with β‐catenin. Our findings demonstrate that ethanol activates NF‐κB1 to upregulate *Traip* expression. TRAIP directly interacts with β‐catenin via its CC domain and directly ubiquitinates β‐catenin through its RING domain in a cell‐free system. In cells, TRAIP promotes K48‐linked ubiquitination and degradation of β‐catenin, which is independent of phosphorylation and acts synergistically with the GSK3β/β‐TrCP pathway. This interaction impairs the β‐catenin/FOXO3 antioxidant signaling axis, exacerbating oxidative damage and inflammatory responses in the liver. Importantly, pharmacological stabilization of β‐catenin via SKL2001 alleviated ethanol‐induced oxidative stress, suggesting a potential therapeutic strategy for ALD. These results expand our understanding the role of TRAIP in liver pathophysiology and underscore the interplay between ubiquitination pathways and oxidative stress regulation in ALD.

The elevated expression of TRAIP in both human and murine ALD tissues suggest its involvement in disease progression. Our data align with prior studies implicating TRAIP in stress responses and inflammation, yet its role in hepatic redox homeostasis has been unexplored. The RING domain of TRAIP, known for E3 ubiquitin ligase activity, was critical for β‐catenin degradation, consistent with the established function of TRAIP in modulating protein stability [20, 26]. Notably, the interaction between TRAIP and β‐catenin via its CC domain points to a novel mechanism by which ubiquitination pathways intersect with Wnt/β‐catenin signaling. Our cell‐free ubiquitination assays provide evidence that TRAIP can functions as a direct E3 ligase for β‐catenin, independent of other cellular proteins. This interaction was associated with reduced levels of FOXO3 and its downstream targets (Figure 7D), suggesting a potential impact on cellular defenses against ROS. These findings complement recent work showing that β‐catenin safeguards against alcohol‐induced steatosis by enhancing antioxidant pathways [14, 15], and raise the possibility that TRAIP might act as an upstream negative regulator of this protective axis.

The activation of NF‐κB1 by ethanol and its subsequent induction of *Traip* establish a feed‐forward loop that amplifies oxidative injury. NF‐κB is a well‐characterized mediator of alcohol‐induced inflammation [27, 28], but its role in regulating E3 ligases in ALD is less understood. Our ChIP and luciferase assays confirmed NF‐κB1 binding to the *Traip* promoter, directly linking ethanol exposure to *Traip* upregulation. This mechanism parallels observations in cancer models, where NF‐κB activation promotes ubiquitin ligase expression to degrade tumor suppressors [29]. In ALD, however, targeting of β‐catenin by TRAIP shifts the balance toward oxidative damage. The application of the NF‐κB1 inhibitor EN450 in mice demonstrated that *Traip* transcription was indeed suppressed, which aligns with the cellular experimental results.

Intriguingly, TRAIP overexpression reduced hepatic triglyceride accumulation despite exacerbating oxidative stress and inflammation. This contrasts with TRAIP deficiency, which worsened steatosis but mitigated injury. While our study focused on β‐catenin/FOXO3 signaling, mass spectrometry identified FASN and CPT1a—key enzymes in lipid synthesis and oxidation—as TRAIP interactors. Further experiments confirmed a direct interaction between TRAIP and CPT1a (Figure S12A,B). Notably, in the TRAIP^LSO^ experimental group, CPT1a protein levels were significantly elevated (Figure S12C), which may explain why TRAIP^LSO^ reduces hepatic triglyceride accumulation. Previous studies have reported that β‐catenin regulates lipid metabolism via PPARα and SREBP1c [30, 31, 32], but imply TRAIP may exert dual roles through distinct targets. To further investigate the causal role of CPT1a, we treated both LV‐TRAIP AML12 cells and TRAIP^LSO^ mice (subjected to ethanol gavage and the NIAAA model) with the specific CPT1a inhibitor Etomoxir. In LV‐TRAIP AML12 cells, Etomoxir treatment markedly increased lipid droplet accumulation (Figure S12D), and significantly elevated ROS levels (Figure S12E). In TRAIP^LSO^ mice with alcohol‐associated liver injury, Etomoxir administration resulted in elevated serum ALT and AST levels (Figure S12G,H,M,N), along with increased hepatic TG (Figure S12I,O) and MDA (Figure S12J,P) content, and reduced SOD (Figure S12K,Q). Moreover, genetic knockdown of CPT1a using siRNA (siCPT1a) recapitulated the effects of Etomoxir in vitro, as evidenced by increased lipid droplet accumulation (Nile Red staining) and elevated ROS levels (Figure S12R,S). Metabolomic analysis further revealed that CPT1a inhibition led to accumulation of lipotoxic metabolites (AHexCer, acidic hexosylceramide), which may contribute to the observed increase in oxidative stress (Figure S12U–W). These findings indicate that CPT1a is the primary mediator of the lipid‐lowering effect in this context, whereas the aggravated oxidative stress is driven predominantly by CPT1a‐independent mechanisms, specifically the TRAIP–β‐catenin axis. The exacerbation of oxidative injury following CPT1a inhibition may be attributable, at least in part, to accumulated lipotoxicity resulting from impaired fatty acid oxidation. Future studies should clarify the underlying mechanisms by which TRAIP regulates lipid metabolism. Furthermore, given that our study utilized a female mouse model to align with established protocols and mitigate confounding hormonal variables, and given the higher susceptibility of females to ALD, future work exploring potential sexual dimorphism in the TRAIP‐mediated regulation of β‐catenin and lipid metabolism could provide valuable insights into sex‐specific aspects of ALD pathogenesis. Additionally, we observed that mRNA levels of *Traip* is also upregulated in a mouse high‐fat diet (HFD) model (Figure S13B). This suggests that TRAIP‐mediated β‐catenin degradation and lipid metabolism regulation may represent a shared pathological node across multiple metabolic liver diseases. Future studies should validate the universality of this pathway in NAFLD models, providing a foundation for developing broad‐spectrum therapeutic strategies for liver diseases.

The attenuated inflammation in TRAIP^LKO^ mice raises questions about the role of TRAIP in immune cell crosstalk. As TRAIP was manipulated specifically in hepatocytes, the observed reductions in TNF‐α and IL‐6 likely reflect altered hepatocyte‐derived signals (e.g., DAMPs or lipid mediators) that modulate Kupffer cell or stellate cell activation [33, 34, 35]. Alternatively, β‐catenin stabilization in TRAIP^LKO^ hepatocytes may enhance anti‐inflammatory signaling, as Wnt/β‐catenin activation is known to suppress NF‐κB‐driven cytokine production [36, 37]. This bidirectional regulation between β‐catenin and inflammatory pathways warrants further exploration in cell‐type‐specific models.

The efficacy of SKL2001 in mitigating oxidative stress without affecting steatosis underscores the complexity of targeting β‐catenin in ALD. While β‐catenin agonists show promise in reducing injury, their inability to address lipid accumulation highlights the need for combinatorial therapies. For instance, pairing SKL2001 with agents that enhance fatty acid oxidation (e.g., PPARα agonists) might achieve dual benefits. Comprehensive in vivo efficacy studies—encompassing assessments of fibrosis resolution, survival benefit, and dose‐response relationships across multiple disease models—are essential future steps to rigorously evaluate any potential therapeutic relevance.

There were some limitations to this study. First, while our data demonstrate that TRAIP promotes β‐catenin degradation and downstream changes in FOXO3a, SOD2, GADD45a, and ROS—consistent with the established β‐catenin/FOXO3 antioxidant pathway—formal demonstration of FOXO3 nuclear localization and transcriptional activity would further strengthen the causal link and remains an important direction for future investigation. Second, the mechanistic investigations in this study mainly focused on oxidative stress, and differences in lipid metabolism and inflammation were not explored. However, as shown in Figure 2D, H‐I, 3D, H‐I, and Figure S6A,B, overexpression and knockout of TRAIP affected lipid metabolism and inflammation. The effects on lipid metabolism may have been directly caused by the target proteins FASN and CPT1a, which interact with TRAIP (Figure 5C). FASN is the rate‐limiting enzyme for fatty acid synthesis, and CPT1a is the key enzyme for fatty acid beta oxidation. Although our study provides initial evidence for the involvement of CPT1a in TRAIP^LSO^ mice, the molecular mechanism through which TRAIP modulates CPT1a expression remains to be fully elucidated. Third, in the liver, inflammatory factors and damage are caused by hepatic stellate cells and macrophages rather than hepatocytes. Liver specific overexpression and knockout of TRAIP in mice did not alter any other cellular components of the liver except for hepatocytes. The differences in inflammation that we observed may have been caused altered metabolism in hepatocytes by overexpression and knockout of TRAIP. Further studies are needed to understand how TRAIP affects the inflammatory response in the liver through hepatocyte involvement. Fourth, the current findings on SKL2001 should be interpreted as mechanistic evidence supporting the involvement of the β‐catenin pathway in oxidative stress regulation, rather than as a validated therapeutic strategy. Fifth, the ALD patient cohort in this study is relatively small (n = 18), which may limit the statistical power for detecting modest differences. Therefore, the correlation between TRAIP expression and clinical parameters warrants validation in larger independent cohorts.

In summary, this study suggests that TRAIP contributes to ALD by promoting β‐catenin degradation and modulating FOXO3 signaling, thereby exacerbating oxidative stress and inflammation. Mechanistically, TRAIP directly binds to β‐catenin and catalyzes its K48‐linked ubiquitination and proteasomal degradation, independent of the canonical GSK3β/β‐TrCP pathway. Pharmacological stabilization of β‐catenin with SKL2001 mitigates ethanol‐induced liver oxidative stress damage, suggesting a potential therapeutic approach. These findings provide new insights into the TRAIP/β‐catenin axis in ALD, highlighting a non‐canonical ubiquitination mechanism, and warranting further investigation into its clinical relevance and therapeutic applicability.

## 4 Experimental Section

### 4.1 Clinical and GEO Datasets Analysis

Clinical liver samples were obtained from ALD patients and healthy controls with informed consent under an approved institutional review board protocol at the First Affiliated Hospital of Guangxi Medical University (details in Supplementary Methods). Three publicly available transcriptomic datasets (GSE143318, GSE142530, GSE155907) from the Gene Expression Omnibus (GEO) were integrated and analyzed to assess *TRAIP* mRNA expression in ALD.

### 4.2 Animal Models

All animal procedures were approved by the Institutional Animal Care and Use Committee of Guangxi Medical University. TRAIP^LSO^ and TRAIP^LKO^ mice on a C57BL/6J background were generated and validated (Figures S1 and S2). Acute and chronic alcohol‐associated liver injury was induced in female mice using the well‐established NIAAA chronic‐plus‐binge feeding model, a double gavage model, or a chronic feeding model, as detailed in Supplementary Methods.

### 4.3 Cell Culture and Treatments

AML12, HepG2, and HEK‐293T cells were cultured under standard conditions. Primary mouse hepatocytes were isolated via collagenase IV perfusion and Percoll gradient centrifugation. AML12 cells were treated with ethanol, the β‐catenin agonist SKL2001, the GSK3β inhibitor TWS119, or the β‐TrCP agonist NRX‐103094. Stable TRAIP‑overexpressing (LV‐TRAIP) and TRAIP‑knockdown (shTRAIP) AML12 cell lines were generated using lentiviral infection. siNF‑κB1 and siCPT1a were used for silencing NF‑κB1 and CPT1a. All cell lines were authenticated and tested mycoplasma‑negative. Detailed protocols are provided in the Supplementary Methods.

### 4.4 Molecular and Biochemical Assays

Standard protocols were followed for quantitative real‐time PCR (qRT‐PCR), western blotting, chromatin immunoprecipitation (ChIP), co‐immunoprecipitation (Co‐IP), luciferase reporter assays, immunofluorescence, and assessment of ubiquitination. Cell‐free in vitro ubiquitination assays were performed using purified recombinant proteins to assess the direct E3 ligase activity of Flag‐TRAIP toward HA‐β‐catenin. Detailed procedures, including primer sequences, antibody information, and plasmid constructs, are provided in Tables S1–S3 and Supplementary Methods.

### 4.5 Histology and Clinical Biochemistry

Liver tissues were processed for H&E, Oil Red O staining and immunohistochemical (IHC) staining for Ly6G, F4/80, CD38, CD163, and 4‐HNE. Serum levels of ALT and AST, along with hepatic triglyceride (TG), malondialdehyde (MDA), superoxide dismutase (SOD), glutathione (GSH) and inflammatory cytokines (IL‐6, TNF‐α), were measured using commercial kits or automated analyzers.

### 4.6 Statistical Analysis

Data were analyzed using GraphPad Prism 9, expressed as mean ± standard deviations (SD). Unpaired Student's *t*‐test was used for two‐group comparisons. One‐way ANOVA with Dunnett's multiple comparison test was used for experiments involving a single independent variable. Two‐way ANOVA with appropriate post‐hoc tests (Sidak's or Tukey's) was used for experiments involving two independent variables. Spearman's rank correlation coefficient was used to assess relationships between two continuous variables. For comparisons across three groups, one‐way ANOVA with Dunnett's post‐hoc test was performed. A *P*‐value of less than 0.05 was considered statistically significant, and significance levels in the figures are denoted as follows: *P* < 0.05 (^*^), *P* < 0.01 (^**^), and *P* < 0.001 (^***^). Non‐significant differences (*P* > 0.05) are labeled as “ns”. Detailed outlier exclusion criteria are described in the Supplementary Methods.

## Author Contributions

Z.W., S.H. and G.Y. contributed conceptualization and methodology. Z.W., M.L., L.L., Z.M., W.X., L.M., Q.X., M.Y., S.C. and G.Y. contributed investigation. Z.W., M.L., L.L., S.H. and G.Y. contributed formal analysis. Z.W., Y.C., S.M., S.T., S.H. and G.Y. contributed writing – original draft. Z.W., M.L. and L.L. contributed visualization. S.H. and G.Y. contributed supervision project administration and funding acquisition. All authors contributed data curation and writing – review & editing. The final version of the manuscript was approved by all authors.

## Conflicts of Interest

The authors declare no conflicts of interest.

## Supporting information




**Supporting File**: advs77644‐sup‐0001‐SuppMat.docx.

## Data Availability

The data that support the findings of this study are available from the corresponding author upon reasonable request.
