# Supplementary material for: TRAIP Mediates Alcohol‐Induced Liver Injury through Regulating β‐catenin Ubiquitin Degradation via Direct Interaction
Source: Adv Sci (Weinh). 2026 Sep 11:e21610. Online ahead of print. doi: 10.1002/advs.202521610 (PMC13564194; doi:10.1002/advs.202521610)
Supplement: Supplementary file 1 [file ADVS-9999-e21610-s001.docx]

**Supporting Information for:**

**TRAIP mediates alcohol-induced liver injury through regulating β-catenin ubiquitin degradation via direct interaction**

Zhan Wu^1,2,3,#^, MingJiang Liu^1,2,3,#^, Lijuan Liao^2,3,#^, Zhaohong Mo^1,2,3^, Wenli Xu^2,3^, Linfeng Mao^1,2,3^, Yubing Chen^1,2,3^, Qiuli Xie^1,2,3^, Ming Yao^2,3^, Shilian Chen^2,3^, Stephen Tomlinson^4^, Sebastian Mueller^5*^, Songqing He^1,2,3*^, Guandou Yuan^1,2,3*^

^1^Division of Hepatobiliary Surgery, The First Affiliated Hospital of Guangxi Medical University, Nanning, Guangxi 530021, China.

^2^Key Laboratory of Early Prevention and Treatment for Regional High Frequency Tumor (Guangxi Medical University), Ministry of Education, Nanning, Guangxi 530021, China.

^3^Guangxi Key Laboratory of Immunology and Metabolism for Liver Diseases, Guangxi Medical University, Nanning, Guangxi 530021, China.

^4^Department of Microbiology and Immunology, Medical University of South Carolina, Charleston, SC, United States.

^5^Center for Alcohol Research, University Hospital Heidelberg, Heidelberg, Germany.

^#^These authors contributed equally to this work.

***Correspondence:**

Guandou Yuan, Division of Hepatobiliary Surgery, The First Affiliated Hospital of Guangxi Medical University, NO 6 Shuangyong Road, Nanning, Guangxi 530021, China. Email: dr_yuangd@gxmu.edu.cn

Songqing He, Division of Hepatobiliary Surgery, The First Affiliated Hospital of Guangxi Medical University, NO 6 Shuangyong Road, Nanning, Guangxi 530021, China. Email: dr_hesongqing@163.com

Sebastian Mueller, Center for Alcohol Research, University Hospital Heidelberg, Heidelberg, Germany. Email: sebastian.mueller@urz.uni-heidelberg.de

**Supplementary Methods**

Clinical Samples and GEO Datasets Analysis: Clinical samples were procured from patients diagnosed with ALD and healthy controls at the First Affiliated Hospital of Guangxi Medical University, following an approved institutional review board protocol. Healthy liver tissue was sourced from living liver transplant donors. The study cohort comprised 18 ALD patients and 12 healthy controls. Transcriptomic analysis of ALD and normal non-ALD liver specimens utilized three datasets from the GEO database: GSE143318, GSE142530, and GSE155907. Specifically, GSE143318 (GPL18573 platform) included 7 healthy controls and 13 alcoholic hepatitis (AH) samples. GSE142530 (GPL11154 platform) comprised 10 AH cases and 12 healthy controls. GSE155907 (GPL21290 platform) consisted of 5 AH cases and 4 healthy controls. The "sva" package's battle function was employed to eliminate batch effects and unknown variables. Post-integration, the final dataset included 28 AH cases and 23 controls.

Mice: Custom-developed TRAIP^LSO^ (Stock No. T006165) and TRAIP Flox mice (Stock No. T009716) were obtained from GemPharmatech Co., Ltd. (Jiangsu, China). Alb-Cre mice (Stock No. 003574) were sourced from The Jackson Laboratory. All three strains were on a C57BL/6J background. TRAIP^LSO^ mice were initially constructed for research purposes in other projects (Figure S1A-C). The expression of TRAIP protein in primary liver cells isolated from TRAIP^LSO^ mice was significantly elevated compared with control mice (Figure S1D), confirming successful construction. TRAIP^LKO^ mice were bred by crossing TRAIP^fl/fl^ with Alb-Cre mice (Figure S2A-C). *Traip* mRNA levels in primary liver cells from TRAIP^LKO^ mice were markedly reduced compared to controls (Figure S2D), indicating successful construction. C57BL/6J wild-type mice (Stock No. N000013) were purchased from GemPharmatech Co., Ltd. (Guangdong, China). All animal experiments and human sample collections were approved by the Ethics Committee of Guangxi Medical University (English version: 2024-E0831).

Murine Models of Acute and Chronic Alcohol Exposure: Female TRAIP^LSO^ and littermate wild-type mice (10-12 weeks old), TRAIP^LKO^ and littermate control mice, C57BL/6J wild-type mice combined with SKL2001 (HY-101085, MCE), EN450 (HY-49444, MCE) and TRAIP^LSO^ mice treated with SKL2001 were used to establish the NIAAA model. TRAIP^LSO^ mice treated with Etomoxir (HY-50202A, MCE) were used to establish ethanol gavage and the NIAAA model. For the NIAAA model, mice were fed a liquid control diet for 5 days, followed by 10 days of a 5% liquid alcohol diet (LAF). On the morning of the 16th day, alcohol gavage (31.5% alcohol, 400 μL/20 g) was administered, and after 9 hours, blood and liver samples were collected. For the ethanol gavage model, mice received alcohol gavage every 12 hours (31.5% alcohol, 400 μL/20 g), with blood and liver samples taken 9 hours after the second gavage. For the chronic model, mice were fed a liquid control diet for 3 days, followed by a 1% liquid alcohol diet for 2 days, a 2% liquid alcohol diet for 2 days, a 4% liquid alcohol diet for 3 days, and a 5% liquid alcohol diet for 3 weeks, after which blood and liver samples were collected. The reference feed and alcohol feed were purchased from Trophic Animal Feed High-Tech Co. LTD, China (TP 4030), and prepared according to the instruction manual.

Murine Models of NCD and HFD Exposure: Male C57BL/6 wild-type (WT) aged 8 to 10weeks, were employed in this study. Normal chow diet and high-fat diet were used to establish a model of diet-induced NAFLD for 16 weeks. The reference feed were purchased from Trophic Animal Feed High-Tech Co. LTD, China (LAD3001G and TP23400).

Extraction of primary liver cells from mice: After modeling, the live liver tissue of mice was perfused with pre perfusion solution (D-Hank's) and digested with DMEM medium containing collagenase IV (500ul containing 3000u) to obtain shed cells. The cells were filtered through a sieve, resuspended with 20% percoll, and centrifuged at 4 ℃ and 500g to obtain liver primary cells. The isolated primary liver cells were used for RNA and protein extraction, as well as ROS detection.

qRT-PCR: Total RNA was extracted from liver tissues or cells using Trizol reagent (15596026, Invitrogen). RNA was reverse transcribed into cDNA using a reverse transcription kit (K1691, Thermo Scientific), followed by real-time quantitative PCR using 2 x Taq SYBR Green Master Mix (172-524, BIO-RAD), cDNA, and primers. Primer sequences are listed in Table S1. Target gene expression was normalized to GAPDH, and relative expression levels were calculated using the 2^-∆∆CT method.

Western Blotting: Protease inhibitor tablets (HY-K0011, MCE) were added to RIPA lysate (P0013D, Beyotime) for tissue and cell lysis. Mouse liver tissue or cells were lysed and ground on ice for 15 minutes, vortexed every 5 minutes. After complete lysis, samples underwent ultrasound fragmentation and centrifugation at 12,000 rpm for 10 minutes at 4 ℃. The supernatant was mixed with protein sample buffer, boiled at 95 ℃ for 5 minutes, then loaded onto a 10% polyacrylamide gel and electrophoresed at 60 V for 30 minutes, followed by 110 V. Proteins were transferred to membranes at a constant current of 200 mA, with transfer time adjusted based on target protein size. Membranes were blocked, stained with antibodies, and developed for imaging. Antibodies used are listed in Table S2.

Hematoxylin and Eosin Staining: Liver samples were fixed with 4% paraformaldehyde (G2161, Solarbio) for 48 hours, paraffin-embedded, sectioned, and stained using an H&E Staining Kit (G1120, Solarbio) following the manufacturer's instructions. Sections were observed under a microscope and photographed.

Oil Red O Staining: Liver tissue samples were embedded in optimal cutting temperature compound (4583, Sakura) and stored at −80°C. Frozen tissues were sliced into 10 μm sections using a cryostat (Leica CM1950, USA). Sections were fixed in 4% formaldehyde calcium fixative, washed, rinsed with 60% isopropanol, stained with Oil Red O (G1260, Solarbio), differentiated until the mesenchyme was clear, and counterstained with hematoxylin. Lipid droplet distribution was observed by microscope.

Immunohistochemistry: Immunohistochemistry was performed on paraffin-embedded liver sections using primary antibodies against 4-HNE, Ly6G, F4/80, CD38, and CD163. Antigen retrieval was performed using citrate buffer, and signals were visualized with DAB substrate after HRP-conjugated secondary antibody incubation. Sections were counterstained with hematoxylin and imaged under a light microscope.

Nile Red Staining: Cells were seeded in 24-well plates containing cell slides. Nile Red dye (N8440, Solarbio) was prepared as a working solution at a concentration of 10 μg/mL. Cells were incubated with the dye for 10 minutes at 37 °C in a cell culture incubator, followed by two washes with PBS. Nuclei were counterstained with DAPI, and images were captured using a fluorescence microscope.

Detection of Serum ALT and AST Levels: ALT and AST levels, indicators of liver function, were measured in model mice using a biochemical analyzer. Serum samples (100 μL) were placed on ice, inserted into the analyzer, and detected following the specified protocol.

Detection of TG, MDA, SOD and GSH in Liver Homogenate: Liver homogenate TG (A110-1-1), MDA (A003-1-2), and SOD (A001-3-2) test kits were purchased from Nanjing Jiancheng Biotechnology Research Institute. Mouse liver samples were homogenized with nine volumes of PBS, and the supernatant was diluted to an appropriate concentration for detection. Liver TG, MDA, SOD and GSH (BC1170, Solarbio) contents were calculated based on the absorbance value to protein or liver weight concentration ratio.

Detection of Inflammatory Factors in Liver Homogenate: IL-6 (EMC004) and TNF-α (EMC102a) ELISA test kits were purchased from Neobioscience. Liver samples were homogenized and diluted, then IL-6 and TNF-α levels were measured according to the kit instructions.

Cell Culture and Cell Line Construction: Mouse hepatocyte (AML12, RRID: CVCL_0140) and Human hepatocellular carcinoma cells (HepG2, RRID: CVCL_0027) were obtained from the Cell Bank/Stem Cell Bank, Chinese Academy of Sciences and received in October 2022. Human embryonic kidney 293T (HEK-293T, RRID: CVCL_0063) were purchased from Cellcook Biotech (Guangzhou, China) and received in December 2023. All cell lines used were authenticated by short tandem repeat profiling and tested negative for mycoplasma contamination. AML12 cells were cultured in complete medium (CM-0602, Procell Life Science&Technology Co., Ltd, China) or complete medium with ethanol (50 mM, 100 mM, 200 mM), NRX-103094 (HY-141449, MCE) or TWS119 (HY-10590, MCE) for 24 hours. HEK-293T cells were cultured in Dulbecco’s Modified Eagle Medium (DMEM) (C11330500BT, Gibco) supplemented with 10% fetal bovine serum and 1% penicillin–streptomycin. All cells were incubated at 37 °C with 5% CO2. AML12 cells were transfected with siRNA using lipofectamine 3000 (L3000015, Invitrogen) to silence NF-κB1 and CPT1a. TRAIP-overexpressing lentivirus (GOSL0341204) was used to infect AML12 cells, followed by puromycin selection for one week. TRAIP mRNA and protein levels were detected to confirm stable overexpression.

ChIP and ChIP-qPCR: AML12 cells were seeded into 15-cm dishes, grown to confluency, and collected for ChIP using a SimpleChIP Plus Enzymatic Chromatin IP Kit (Magnetic Beads, # 9005, CST). The procedure included cell culture cross-linking, nucleus preparation, chromatin digestion, immunoprecipitation with NF-κB1 antibody, DNA purification, PCR amplification, and electrophoretic imaging. For ChIP-qPCR, AML12 cells transfected with siNC and siNF-κB1 were subjected to ChIP, and qPCR was performed using primer B3.

Transcription Factor Binding Site Luciferase Reporter: A fragment containing the predicted transcription factor binding site (base sequence "GGGACTCCTC") in the TRAIP promoter region, along with 368 bp upstream and downstream, was inserted into the pGL4.17 [luc2/Neo] vector, yielding B-WT and B-Mut plasmids. AML12 cells were co-transfected with various combinations of siNC, siNF-κB1, B-WT, and B-Mut. After 24 hours, luciferase activity was detected using a Dualuc® Firefly&Renilla Assay Kit (F6075S, UElandy).

Co-IP: Total protein was extracted from AML12 cells transfected with LV-TRAIP. Cells were lysed in IP lysate with protease inhibitors, centrifuged, and the supernatant was used for Co-IP with target antibody and control IgG. Protein complexes were captured using protein A/G magnetic beads, washed, and detected by western blot.

Mass Spectrometry: Co-IP was performed on LV-TRAIP AML12 cell extracts treated with ethanol (200 mM). Protein complexes obtained with TRAIP antibody were analyzed by mass spectrometry at Hangzhou Jingjie Biotechnology Company.

Lipidomics: LV-TRAIP AML12 cells were treated with ethanol (200 mM) with or without Etomoxir (5 μM) for 24 h. Cell culture supernatants (n=6/group) were collected for non-targeted quantitative lipidomics analysis at Wuhan MetWare Biotechnology Co., Ltd.

TOP/FOP Luciferase Report: AML12, TRAIP Con AML12, and LV-TRAIP AML12 cells were seeded into 12 well plates. Cells were transfected with TOP or FOP Flash plasmid and β-gal as an internal. reference After 24 hours, luciferase and β-gal activities were measured, and calibrated luciferase activity was calculated.

ROS Flow Detection: Primary hepatocytes, HepG2, AML12, TRAIP Con AML12, and LV-TRAIP AML12 cells were treated with anhydrous ethanol, SKL2001, Etomoxir or siCPT1a for 24 hours. ROS levels were detected using a ROS red fluorescence assay kit (CA1420, Solarbio) and flow cytometry.

Plasmids: Full-length, truncated and RING domain mutant (C7A/C10A) TRAIP plasmids were constructed using the pCH-3×FLAG expression vector. Full-length and truncated β-catenin plasmids were constructed using the pcDNA3.0-3×HA expression vector. Plasmid information is provided in Table S3.

Immune Co-localization: Immunofluorescence staining for TRAIP and β-catenin was performed to evaluate their co-localization. Cells were fixed, permeabilized, blocked, and incubated with primary and secondary antibodies. Slides were mounted with DAPI, and confocal images were captured.

Protein Domain Binding Analysis: Cells were co-transfected with the Flag-TRAIP WT plasmid and the HA-β-catenin WT, HA-β-catenin D1, HA-β-catenin D2, and HA-β-catenin D3 plasmid. Cells were co-transfected with the HA-β-Catenin WT plasmid and the Flag-TRAIP WT, Flag-TRAIP D1, Flag-TRAIP D2, Flag-TRAIP D3, Flag-TRAIP D4, Flag-TRAIP D5, Flag-TRAIP D6, or Flag-TRAIP D7 plasmid, and pull-down assays were performed to analyze protein domain interactions. Each pair of plasmids was co-transfected into HEK-293T cells in a 10-cm culture dish, followed by cell lysis using IP lysis buffer. A small amount of cell lysis buffer was retained for WB, while the rest was used for pulldown. The group co-transfected with Flag antibody pull-down Flag-TRAIP WT plasmid and HA-β-catenin WT, HA-β-catenin D1, HA-β-catenin D2, HA-β-catenin D3 plasmids, and co-transfected with HA antibody pull-down HA-β-catenin WT plasmid and Flag-TRAIP WT, Flag-TRAIP D1, Flag-TRAIP D2, Flag-TRAIP D3, Flag-TRAIP D4, Flag-TRAIP D5, Flag-TRAIP D6, Flag-TRAIP D7 plasmids.

In Vivo Ubiquitination Assay: For endogenous ubiquitination, AML12 cells infected with Con or LV-TRAIP viruses were treated with MG132, lysed, and Co-IP was performed with β-catenin antibody. For exogenous ubiquitination, HEK-293T cells were transfected with plasmids, treated with MG132, and Co-IP was performed with His_6_ antibody. Protein complexes were detected with anti-ubiquitin, anti-HA, K48-linked ubiquitin or K63-linked ubiquitin antibodies.

In Vitro Ubiquitination Assay: Recombinant Flag-tagged WT or RING domain mutant (C7A/C10A) TRAIP and HA-tagged β-catenin were expressed in HEK293T cells and purified using BeaverBeads Anti-Flag (22206-1, Beaver Biomedical Engineering Co., Ltd.) or Anti-HA Magnetic Beads (P2121, Beyotime), followed by competitive elution with 3xFlag peptides (HY-P0319, MCE) or 3xHA peptides (HY-P5491, MCE), respectively. For in vitro ubiquitination assays, purified HA-β-catenin was incubated with E1 (UBE-024, UB-biotech), E2 (UBE-627, UB-biotech), His_6_-Ubiquitin peptide (UB-530, UB-biotech), ATP (B-20, UB-biotech), and purified Flag-TRAIP (WT or mutant) in ubiquitination buffer (B-01, UB-biotech) at 37°C for 2 hours. The reaction was terminated by adding SDS-PAGE loading buffer and boiling for 5 minutes. The ubiquitination products were analyzed by immunoblotting with anti-HA antibody to detect β-catenin ubiquitination and anti-Flag antibody to confirm TRAIP input. Omission of individual components served as negative controls.

Statistical Analysis: Data were analyzed using GraphPad Prism 9, expressed as mean ± standard deviations (SD). Unpaired Student's t-test was used for two-group comparisons. One-way ANOVA with Dunnett’s multiple comparison test was used for experiments involving a single independent variable. Two-way ANOVA with appropriate post-hoc tests (Sidak's or Tukey's) was used for experiments involving two independent variables. Spearman's rank correlation coefficient was used to assess relationships between two continuous variables. For comparisons across three groups, one-way ANOVA with Dunnett's post-hoc test was performed. A *P*-value of less than 0.05 was considered statistically significant, and significance levels in the figures are denoted as follows: *P* < 0.05 (*), *P* < 0.01 (**), and *P* < 0.001 (***). Non-significant differences (*P* > 0.05) are labeled as “ns”. Outliers were identified using Grubbs' test (α = 0.05). Exclusion criteria were pre‑established and applied blindly and uniformly across all experimental groups. Data points identified as outliers were excluded from the final statistical analysis, and a summary of excluded outliers for each experiment is provided in Table S4. Raw data, including all excluded values, are provided in the Source Data file.

**Supplemental tables**

**Table S1. Primers for qRT-PCR**

| **Name** | **Forward primers** | **Reverse primers** |
| --- | --- | --- |
| Human-*TRAIP* | 5’-ACAGTGCCGAATCCAGGTTG-3’ | 5’-CGTTTCTCCTTGTCTTTCTGGG-3’ |
| Human-*GAPDH* | 5’-CTGGGCTACACTGAGCACC-3’ | 5’-AAGTGGTCGTTGAGGGCAATG-3’ |
| Mouse-*Traip* | 5’-ACTTAACAGGCACTGGTCGG-3’ | 5’-GCCTTCCCTCTCTTCATCCG-3’ |
| Mouse-*Gapdh* | 5’-CCCACTAACATCAAATGGGG-3’ | 5’-CCTTCCACAATGCCAAAGTT-3’ |
| Mouse-*Nfkb1* | 5’-ATGGCAGACGATGATCCCTAC-3’ | 5’-CGGAATCGAAATCCCCTCTGTT-3’ |
| Mouse-B1 | 5’-TTTGGGTAGCGGTTGATGCT-3’ | 5’- CGCTCAGCTCGGGATAGTTT-3’ |
| Mouse-B2 | 5’-TTTTGGGTAGCGGTTGATGC-3’ | 5’-TCGTGGGATTTTTAGCGTCCA-3’ |
| Mouse-B3 | 5’-GGAGGTCACCTTCTCCAGTG-3’ | 5’-TCCTATAGTGGTTGGCCCTGA-3’ |

**Table S2. Antibodies**

| **Name** | **Supplier** | **Cat no.** |
| --- | --- | --- |
| TRAIP | Proteintech | 10332-1-AP |
| GAPDH | Proteintech | 10494-1-AP |
| NF-kB1 | Cell Signaling Technology | #13586 |
| β-Catenin | BD Biosciences | 610154 |
| IgG | Cell Signaling Technology | #2729 |
| normal mouse IgG | Santa Cruz Biotechnology | sc-2025 |
| Flag-tag | Gigma-Aldrich | F1804 |
| HA-tag | Abcam | ab9110 |
| Ubiquitin | Invitrogen | PA5-102555 |
| His-tag | Proteintech | 66005-1-Ig |
| 4-HNE | Servicebio | GB150073 |
| Ly6G | CST | 87048S |
| F4/80 | Abcam | ab6640 |
| CD38 | Affinity | #DF6551 |
| CD163 | Proteintech | 16646-1-AP |
| FOXO3a | Proteintech | 10849-1-AP |
| SOD2 | Proteintech | 24127-1-AP |
| GADD45a | Abcam | Ab180768 |
| K48-linkage Specific Polyubiquitin | Cell Signaling Technology | #8081 |
| K63-linkage Specific Polyubiquitin | Cell Signaling Technology | #5621 |
| Phospho-β-catenin (Ser33/37/Thr41) | Cell Signaling Technology | 9561S |
| β-TrCP | Cell Signaling Technology | 4394S |
| GSK3β | Proteintech | 22104-1-AP |
| CPT1a | Proteintech | 15184-1-AP |
| HRP-conjugated Goat Anti-Rabbit IgG(H+L) | Proteintech | SA00001-2 |
| HRP-conjugated Goat Anti-Mouse IgG(H+L) | Proteintech | SA00001-1 |
| IPKine™ HRP Mouse Anti-Rabbit IgG LCS | Abbkine | A25022 |
| Goat Anti-Mouse IgG (H+L) Fluor488-conjugated | Affinity Biosciences | S0017 |
| Goat Anti-Rabbit IgG (H+L) CY3-conjugated | Affinity Biosciences | S0011 |

**Table S3. Plasmids**

| **Name** | **Tag** | **Amino Acid** | **Expected MW (KDa)** |
| --- | --- | --- | --- |
| TRAIP-WT | Flag | Full-length | 56 |
| TRAIP-D1 | Flag | 53-470aa | 49 |
| TRAIP-D2 | Flag | 1-70aa, 177-470aa | 43 |
| TRAIP-D3 | Flag | 1-201aa, 280-470aa | 46 |
| TRAIP-D4 | Flag | 1-280aa, 382-470aa | 44 |
| TRAIP-D5 | Flag | 1-382aa, 400-470aa | 53 |
| TRAIP-D6 | Flag | 1-410aa | 48 |
| TRAIP-D7 | Flag | 1-461aa | 54 |
| TRAIP-C7A/C10A | Flag | Full-length C7A/C10A | 56 |
| β-catenin-WT | HA | Full-length | 95 |
| β-catenin-D1 | HA | 1-134aa | 18 |
| β-catenin-D2 | HA | 135-664aa | 61 |
| β-catenin-D3 | HA | 665-781aa | 16 |

**Table S4. Summary of excluded outliers**

| **Figure** | **Group** | **Number of outliers excluded** |
| --- | --- | --- |
| Figure 2C | TRAIP^LSO^ EtOH-fed | 1 |
| Figure 2D | WT Pair-fed | 1 |
| Figure 2D | TRAIP^LSO^ Pair-fed | 1 |
| Figure 2E | WT Pair-fed | 1 |
| Figure 2E | TRAIP^LSO^ Pair-fed | 1 |
| Figure 2E | WT EtOH-fed | 2 |
| Figure 2E | TRAIP^LSO^ EtOH-fed | 1 |
| Figure 2F | TRAIP^LSO^ EtOH-fed | 1 |
| Figure 2H | WT Pair-fed | 3 |
| Figure 2H | TRAIP^LSO^ Pair-fed | 3 |
| Figure 2H | WT EtOH-fed | 3 |
| Figure 2H | TRAIP^LSO^ EtOH-fed | 3 |
| Figure 2I | TRAIP^LSO^ Pair-fed | 2 |
| Figure 2I | WT EtOH-fed | 2 |
| Figure 2I | TRAIP^LSO^ EtOH-fed | 1 |
| Figure 3C | WT Pair-fed | 1 |
| Figure 3C | TRAIP^LKO^ EtOH-fed | 1 |
| Figure 3D | TRAIP^LKO^ EtOH-fed | 1 |
| Figure 3E | WT EtOH-fed | 1 |
| Figure 3F | WT EtOH-fed | 1 |
| Figure 3F | TRAIP^LKO^ EtOH-fed | 1 |
| Figure 3H | WT Pair-fed | 1 |
| Figure 3H | TRAIP^LKO^ Pair-fed | 1 |
| Figure 3H | WT EtOH-fed | 3 |
| Figure 3H | TRAIP^LKO^ EtOH-fed | 4 |
| Figure 3I | WT EtOH-fed | 1 |

**Supplemental Figures**

**Supplemental figure 1.** Constructs used to induce TRAIP^LSO^ in mice. A) Construct design used to induce TRAIP^LSO^ in mice. B) Primers used for mouse genotyping. C) Mouse genotyping results. D) TRAIP protein levels in TRAIP^LSO^ mice. P: Positive control, W: WT control, B: Blank, Ki/ki: 41, 47, 49, Ki/Wt: 43, 44, 46, 48, Wt/wt: others.

**Supplemental figure 2.** Constructs used to induce TRAIP^LKO^ in mice. A) Construct design. B) Primers used for mouse genotyping. C) Mouse genotyping results. D) *Traip* mRNA levels in TRAIP^LKO^ mice. Data represent mean±SD, unpaired t-test was applied; ****p* < 0.001.

**Supplemental figure 3.** Spontaneous hepatic lipid accumulation in aged TRAIP^LSO^ mice. A) Body weight of WT and TRAIP^LSO^ mice at 6m, 9m and 12m. B) Liver weight of WT and TRAIP^LSO^ mice at 6m, 9m and 12m. C, D) H&E and oil red O staining of liver tissues from WT and TRAIP^LSO^ mice at 6m, 9m and 12m, Scale bar=100 μm. Data represent mean±SD, unpaired t-test was applied; ****p* < 0.001.m: month.

**Supplemental figure 4.** Ethanol exposure upregulates TRAIP expression. A, B) TRAIP mRNA and protein levels in AML12 cells treated with ethanol (NC, 50mM, 100mM, 200mM) for 24h (n=3/group). C) TRAIP protein levels in HepG2 cells treated with ethanol (100mM) for 24h (n=3/group). D-F) Correlation analysis of *TRAIP* mRNA levels and TBIL, ALB and CHE levels in serum from healthy control (n=9) and patients with ALD (n=18) (Total n=27). Data represent mean±SD, unpaired t-test and spearman correlation analysis were applied. **P* < 0.05, ***P* < 0.01, ****P* < 0.001, ns: not significant.

**Supplemental figure 5.** TRAIP^LSO^ mouse ethanol gavage (1d) and chronic feeding (4w) models. A) H&E staining of liver tissues from WT and TRAIP^LSO^ mice subjected to ethanol gavage (1d), Scale bar=100 μm. B, C) Serum ALT and AST levels in WT and TRAIP^LSO^ mice subjected to ethanol gavage (1d). D) H&E staining of liver tissues from mice WT and TRAIP^LSO^ subjected to chronic feeding (4w), Scale bar=100 μm. E, F) Serum ALT and AST levels in WT and TRAIP^LSO^ mice subjected to chronic feeding (4w). Data are presented as mean ± SD. P values were calculated by two-way ANOVA. **P* < 0.05, ***P* < 0.01, ****P* < 0.001, ns: not significant.

**Supplemental figure 6.** TRAIP regulates neutrophil infiltration and macrophage polarization in ALD. A) IHC of liver tissues from WT and TRAIP^LSO^ mice in the NIAAA model for Ly6G, F4/80, CD38, and CD163, Scale bar=100 μm. B) IHC of liver tissues from WT and TRAIP^LKO^ mice in the NIAAA model for Ly6G, F4/80, CD38, and CD163, Scale bar=100 μm.

**Supplemental Figure 7.** NF-κB1 activates *Traip* transcription. A) JASPAR database prediction of transcription factors associated with *Traip*. B, C) NF-κB1 mRNA and protein levels in AML12 cells treated with ethanol (100mM) for 24h (n=3/group). D) NF-κB1 protein levels in HepG2 cells treated with ethanol (100mM) for 24h (n=3/group). E) NF-κB1 protein levels in WT mice treated with paired-fed and EtOH-fed (n=3/group). F, G) *Nfkb1* and *Traip* mRNA levels in AML12 cells treated with siNF-κB1 for 24h (n=3/group). H) NF-κB1 protein levels in AML12 cells treated with siNF-κB1 for 24h (n=3/group). I, J) *Traip* promoter DNA fragment pulled down by NF-κB1. (Note: Input, the histone H3 antibody, and the RPL30 DNA fragment serve as positive controls, while IgG functions as the negative control. B1, B2, and B3 represent the DNA fragments amplified by three primer sets spanning the upstream and downstream regions of the *Traip* promoter.) K) H&E staining of liver tissues from WT, WT + EN450 (NF-κB1 inhibitor,10mg/kg or 25mg/kg) mice subjected to paired-fed and EtOH-fed, Scale bar=100 μm. L, M) Serum ALT (L,n=7-8/group) and AST(n=7-8/group) levels. N) The mRNA of *Traip* levels were presented using the same grouping as in panel (A). (n=6/group) O) The protein level of NF-κB1 and TRAIP were presented using the same grouping as previous(n=3/group). Data are presented as mean ± SD. P values were calculated by one-way ANOVA or unpaired t-test as appropriate. **P* < 0.05, ***P* < 0.01, ****P* < 0.001, ns: not significant.

**Supplemental figure 8.** Key mass spectrometry features of β-catenin. A) β-catenin peptide segments. B) Mass-to-charge ratio and intensity of β-Catenin peptide segment.

**Supplemental Figure 9.** The ubiquitination degradation of β-catenin relies on the TRAIP RING structure. A) β-catenin protein levels in AML12 cells treated with ethanol (100mM) for 24h (n=3/group). B) β-catenin protein levels in HepG2 cells treated with ethanol (100mM) for 24h (n=3/group). C) β-catenin protein levels in a mouse NIAAA model (n=3/group). D) TRAIP protein levels in Control and LV-TRAIP AML12 cells treated with ethanol (100mM) for 24h (n=3/group). E) β-TrCP and GSK3β protein levels in HepG2 cells treated with ethanol (100mM) for 24h (n=4/group). F) β-TrCP and GSK3β protein levels in a mouse NIAAA model (n=3/group). G) Quantification of ubiquitin protein levels in (Figure 6D) (n=3/group). H) Quantification of HA-β-catenin protein levels in (Figure 6E) (n=3/group). I) Analysis of exogenous ubiquitination for full-length TRAIP, RING domain-deleted TRAIP, and CC domain-deleted TRAIP. J) Quantification of K48-linked Ubiquitin protein levels in (Figure 6G) (n=3/group). K) Quantification of K63-linked Ubiquitin protein levels in (Figure 6H) (n=3/group). L, M) Co-IP of Flag-TRAIP C7A/C10A and HA-β-catenin (n=3/group). N) β-catenin and FOXO3a protein levels in TRAIP^LKO^ primary hepatocytes treated with ethanol and Flag-TRAIP WT or Flag-TRAIP C7A/C10A mutant (100mM) for 24h (n=3/group). O) ROS levels in TRAIP^LKO^ primary hepatocytes treated with ethanol and Flag-TRAIP WT or Flag-TRAIP C7A/C10A mutant (100mM) for 24h (n=3/group). P) β-TrCP protein levels in ethanol-treated AML12 cells with or without NRX-103094 (25uM) for 24h (n=4/group). Data represent mean ± SD. P values were calculated by one-way ANOVA, two-way ANOVA or unpaired t-test as appropriate. **p* < 0.05; ***p* < 0.01; ****p* < 0.001, ns: not significant.

**Supplemental Figure 10.** ROS results are consistent across primary hepatocytes, HepG2, and AML12 Cells. A). ROS activity levels in HepG2 cells treated with ethanol and SKL2001 for 24h. B) ROS activity in Primary hepatocytes of WT and TRAIP^LSO^ mice in alcohol gavage model (n=3/group). C) Primary hepatocytes β-catenin and TRAIP protein levels of WT and TRAIP^LSO^ mice in alcohol gavage model (n=3/group). Data represent mean ± SD. P values were calculated by one-way ANOVA or two-way ANOVA or as appropriate. **p* < 0.05; ***p* < 0.01; ****p* < 0.001.

**Supplemental Figure 11.** SKL2001 respectively replied to the phenotypes of TRAIP^LSO^ mice. A) H&E staining of liver tissues from TRAIP^LSO^ EtOH-fed mice treated with control and SKL2001, Scale bar=100 μm. B, C) Serum ALT and AST levels in TRAIP^LSO^ mice were presented using the same grouping as in panel (A). Data represent mean ± SD, unpaired t-test was applied. **p* < 0.05; ***p* < 0.01; ****p* < 0.001.

**Supplemental Figure 12.** TRAIP interacts with CPT1a and regulates lipid metabolism. A, B) Co-IP of TRAIP and β-catenin (n=3/group). C) CPT1a protein levels of WT and TRAIP^LSO^ mice in NIAAA model (n=3/group). D, E) Nile red staining and ROS activity levels in LV-TRAIP AML12 cells treated with ethanol and Etomoxir (5uM) for 24h (n=3/group). F) H&E staining of liver tissues from TRAIP^LSO^ mice treated with control and Etomoxir (1mg/kg) subjected to ethanol gavage (1d), Scale bar=100 μm. G, H) Serum ALT and AST levels from TRAIP^LSO^ mice treated with control and Etomoxir subjected to ethanol gavage (1d) (n=6/group). I-K) Liver TG, MDA and SOD levels from TRAIP^LSO^ mice treated with control and Etomoxir subjected to ethanol gavage (1d) (n=6/group). L) H&E staining of liver tissues from TRAIP^LSO^ EtOH-fed mice treated with control and Etomoxir (1mg/kg) (NIAAA), Scale bar=100 μm. M, N) Serum ALT and AST levels from TRAIP^LSO^ EtOH-fed mice treated with control and Etomoxir (NIAAA) (n=9/group). O-Q) Liver TG, MDA and SOD levels from TRAIP^LSO^ EtOH-fed mice treated with control and Etomoxir (NIAAA) (n=9/group). R, S) Nile red staining and ROS activity levels in LV-TRAIP AML12 cells treated with ethanol and siCPT1a for 24h. T) CPT1a protein levels in AML12 cells treated with siCPT1a for 24h (n=3/group). U) Volcano plot of lipidomics analysis in LV-TRAIP AML12 cells treated with ethanol and Etomoxir (5uM) for 24h (n=6/group). Red and green dots represent significantly upregulated and downregulated lipids, respectively (VIP > 1, P < 0.05). V) Violin plot of AHexCer (O-16:4)16:1;2O/17:0;O levels (n=6/group). W) KEGG pathway enrichment analysis of differentially accumulated lipids. Data represent mean ± SD. P values were calculated by one-way ANOVA or unpaired t-test as appropriate. **p* < 0.05; ***p* < 0.01; ****p* < 0.001.

**Supplemental Figure 13.** *Traip* mRNA levels is upregulated in a mouse HFD model. A) H&E staining of liver tissues from WT HFD-fed mice, Scale bar=100 μm. B) *Traip* mRNA levels of WT mice in HFD model (n=5-8/group). Data represent mean ± SD, unpaired t-test was applied. ***p* < 0.01.
